# Poxvirus attack of antiviral defense pathways unleashes an effector-triggered NF-κB response

**DOI:** 10.1126/science.adw4937

**Published:** 2026-02-12

**Authors:** Brenna C. Remick, Joshua Q. Mao, Andrew G. Manford, Ami D. Gutierrez-Jensen, Allon Wagner, Michael Rape, Grant McFadden, Masmudur M. Rahman, Moritz M. Gaidt, Russell E. Vance

**Affiliations:** 1 Department of Molecular and Cell Biology, University of California, Berkeley; Berkeley, CA, USA.; 2 School of Life Sciences and Biodesign Institute, Arizona State University; Tempe, AZ, USA.; 3 Department of Electrical Engineering and Computer Sciences, University of California, Berkeley; Berkeley, CA, USA.; 4 Center for Computational Biology, University of California, Berkeley; Berkeley, CA, USA.; 5 Research Institute of Molecular Pathology, Vienna BioCenter; Vienna, Austria.; 6 Cancer Research Laboratory, University of California, Berkeley; Berkeley, CA, USA.; 7 Howard Hughes Medical Institute, University of California, Berkeley; Berkeley, CA, USA.

## Abstract

Effector-triggered immunity (ETI) is a form of pathogen sensing that involves detection of pathogen-encoded virulence factors or “effectors”. To discover ETI pathways in mammals, we developed a screening approach in which we expressed individual virulence factors in a human monocyte cell line and assessed transcriptional responses by RNA-seq. We identified a poxvirus effector, myxoma virus M3.1, which elicited an antiviral NF-κB response. NF-κB was unleashed by an ETI pathway that sensed M3.1 attack of two antiviral complexes: ZAP and TBK1. NF-κΒ activation occurred because the proteins inhibited by M3.1—N4BP1, ZC3H12A, and TBK1—are negative regulators of NF-κB. Our study established a systematic approach for the discovery of ETI pathways, and the results illustrated how negative regulators of immune responses may function in pathogen sensing.

The mammalian innate immune system senses pathogens by detecting conserved molecular motifs called )pathogen-associated molecular patterns (PAMPs) (1). PAMPs such as lipopolysaccharide and flagellin bind to germline-encoded receptors called pattern-recognition receptors (PRRs) to elicit immune responses referred to as PAMP-triggered immunity (PTI). Sensing of PAMPs is an effective means to differentiate self from non-self, but cannot easily discriminate between pathogens and harmless microbes, as PAMPs are present on both.

A distinguishing feature of pathogens is the expression of virulence factors or “effectors” (2), which pathogens employ to attack key host defenses. Hosts have evolved to “guard” key pathways commonly targeted by virulence factors, such that protective immune responses are initiated when these pathways are disrupted (3, 4). Immune sensing of virulence factor activities in host cells is referred to as effector-triggered immunity (ETI) (3, 5). In contrast to PTI, ETI is inherently pathogen-specific, as innocuous microbes do not deliver virulence factors into host cells. The concept of ETI originated in studies of plant immunity (3, 5), but a few examples of ETI have been described in animals as well (6–8). However, it is unclear whether these examples are representative of a more ubiquitous—and perhaps underappreciated—form of pathogen detection.

Investigations of ETI are complicated by the evolutionary arms races between hosts and pathogens, which result in layers of host immune responses and pathogen counterattacks (8, 9). This interplay is captured by the “zig-zag” model of plant immunity (3), in which sensing of PAMPs by PRRs elicits a host immune response which is then blocked by pathogen virulence factors. In turn, hosts evolve mechanisms to detect virulence factors or their activities, resulting in ETI. Pathogens may then evolve additional virulence factors that suppress ETI, giving rise to a “zig-zag” pattern of alternating susceptibility and resistance. Consequently, pathogens that are well-adapted to a host may fail to elicit detectable ETI responses, yet ETI pathways may still exist and confer protection in other contexts.

In this study, we sought to identify pathogen virulence factors that elicit ETI responses in human cells. We screened a library of open reading frames (ORFs) from myxoma virus (MYXV) (10), a rabbit-adapted poxvirus lethal to European rabbits (11). Like other poxviruses, MYXV harbors a large, double-stranded DNA (dsDNA) genome encoding dozens of virulence factors (12, 13). MYXV does not infect humans or mice, but it can infect certain human cancer cell lines (14). We reasoned that virulence factors from MYXV may be more likely to trigger ETI responses in human cells than those from a well-adapted pathogen. By screening individual MYXV virulence factors, we anticipated we could identify ETI pathways that might be blocked by additional virulence factors during infection with an intact virus. Our results illustrate that our approach is likely to be broadly applicable for the discovery of other ETI pathways in humans that pathogens may have evolved to evade in order to be infectious.

## Results

### Systematic virulence factor screening identifies MYXV M3.1 as an inducer of NF-κB signaling

**Fig. 1.**
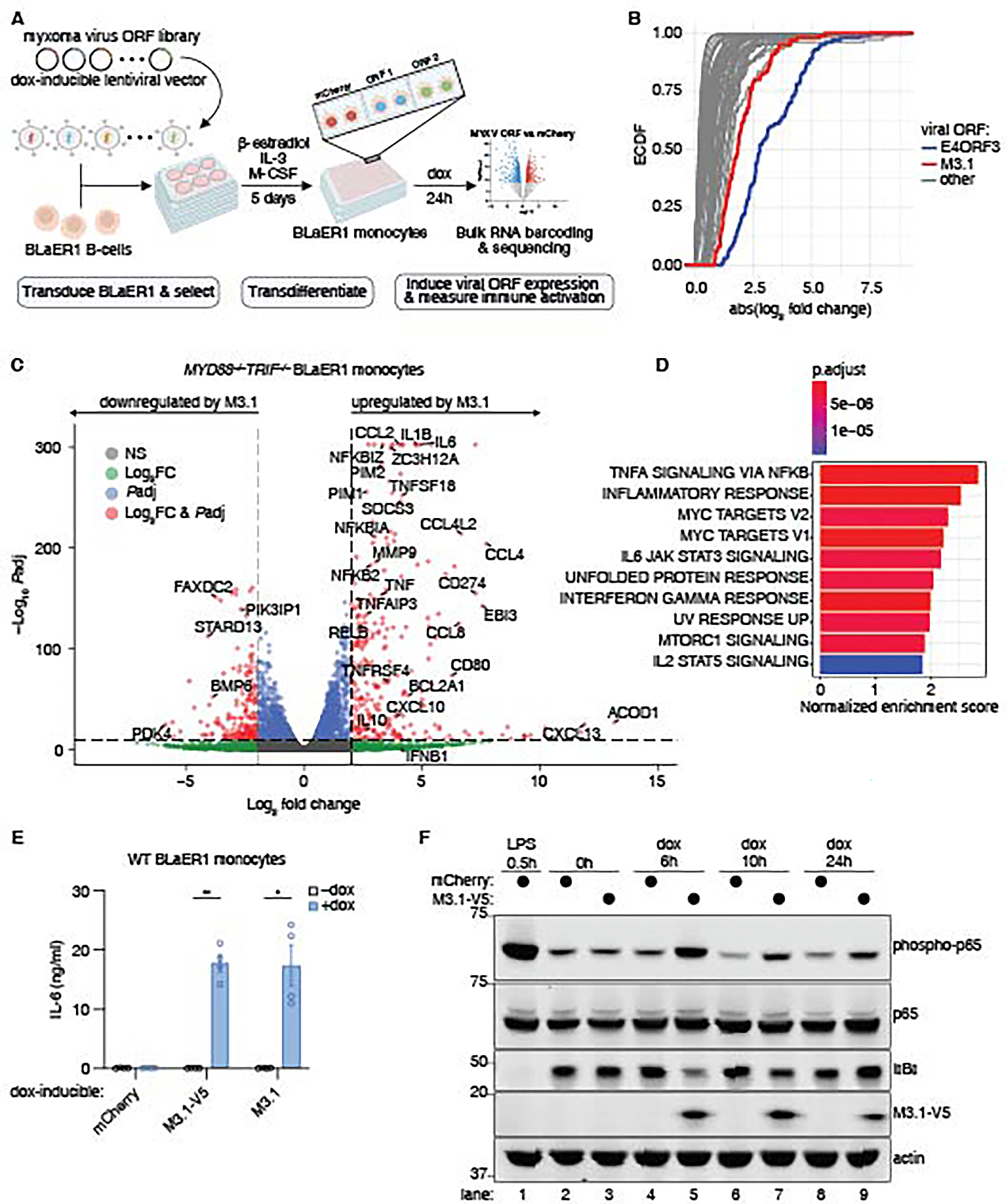
MYXV M3.1 induces NF-κB signaling in human monocytes. (**A**) Schematic of the arrayed virulence factor screening approach. Created with BioRender.com. (**B**) For each viral ORF, DESeq2 was used to identify differentially expressed host genes compared to the mCherry control. The empirical cumulative distribution function (ECDF) of the absolute fold change was plotted for the top 100 genes with the lowest *P*adj value for each ORF. (**C**) Volcano plot of differentially expressed genes in *MYD88*^*–/–*^*TRIF*^*–/–*^ BLaER1 monocytes treated with doxycycline for 24 hours to induce expression of M3.1 or mCherry. Data from three independent experiments. Dashed lines indicate a log_2_FC cutoff of ±2 and a *P*adj cutoff of 10e-10. (**D**) MSigDB Hallmark gene sets enriched in M3.1-expressing *MYD88*^*–/–*^*TRIF*^*–/–*^ BLaER1 monocytes in (C). (**E**) IL-6 secreted by BLaER1 monocytes treated with doxycycline for 24 hours. Each data point represents an independent experiment; bars indicate mean ± SEM of four independent experiments. (**F**) BLaER1 monocytes expressing doxycycline-inducible mCherry or M3.1 were treated with doxycycline or LPS (200 ng/ml) for the indicated times. Lysates were immunoblotted as indicated. Images are representative of three independent experiments. * *P* < 0.05; ** *P* < 0.01, tested by unpaired t-test with Welch’s correction.

We expressed an arrayed and doxycycline-inducible library of MYXV ORFs in human BLaER1 monocytes (15) (Fig. 1A). Our library included 117 MYXV ORFs (10), mCherry as a negative control, and adenovirus E4ORF3, a virulence factor that induces a type I interferon (IFN) response (16), as a positive control. For each ORF-expressing cell line (Data S1), we performed bulk RNA barcoding and sequencing (17) to identify MYXV virulence factors that elicited host transcriptional responses relative to mCherry (Fig. 1B). Adenovirus E4ORF3 induced a robust type I IFN response (Fig. S1). The majority of MYXV ORFs did not elicit notable transcriptional responses. However, several MYXV ORFs induced differential host gene expression, which we characterized by gene set enrichment analysis (Fig. S2). Expression of the MYXV M003.1 gene (encoding the M3.1 protein) induced a strong NF-κB-like transcriptional response in BLaER1 monocytes (Fig. S2B).

We confirmed that the transcriptional response induced by M3.1 was independent of trace TLR ligands (e.g., LPS, lipoproteins) potentially present in the media by expressing M3.1 in *MYD88*^*–/–*^*TRIF*^*–/–*^ BLaER1 monocytes and performing RNA-seq. Again, we observed robust induction of inflammatory cytokines and chemokines by M3.1, characteristic of an NF-κB-mediated immune response (Fig. 1C-D). We confirmed IL-6 protein induction by M3.1, which was unaffected by a C-terminal V5 epitope tag on M3.1 (Fig. 1E). Moreover, we observed IκBα degradation and sustained p65 phosphorylation in M3.1-expressing BLaER1 monocytes (Fig. 1F), providing biochemical evidence that the NF-κB signaling pathway is activated by M3.1.

### Identification of M3.1 cellular targets

**Fig. 2.**
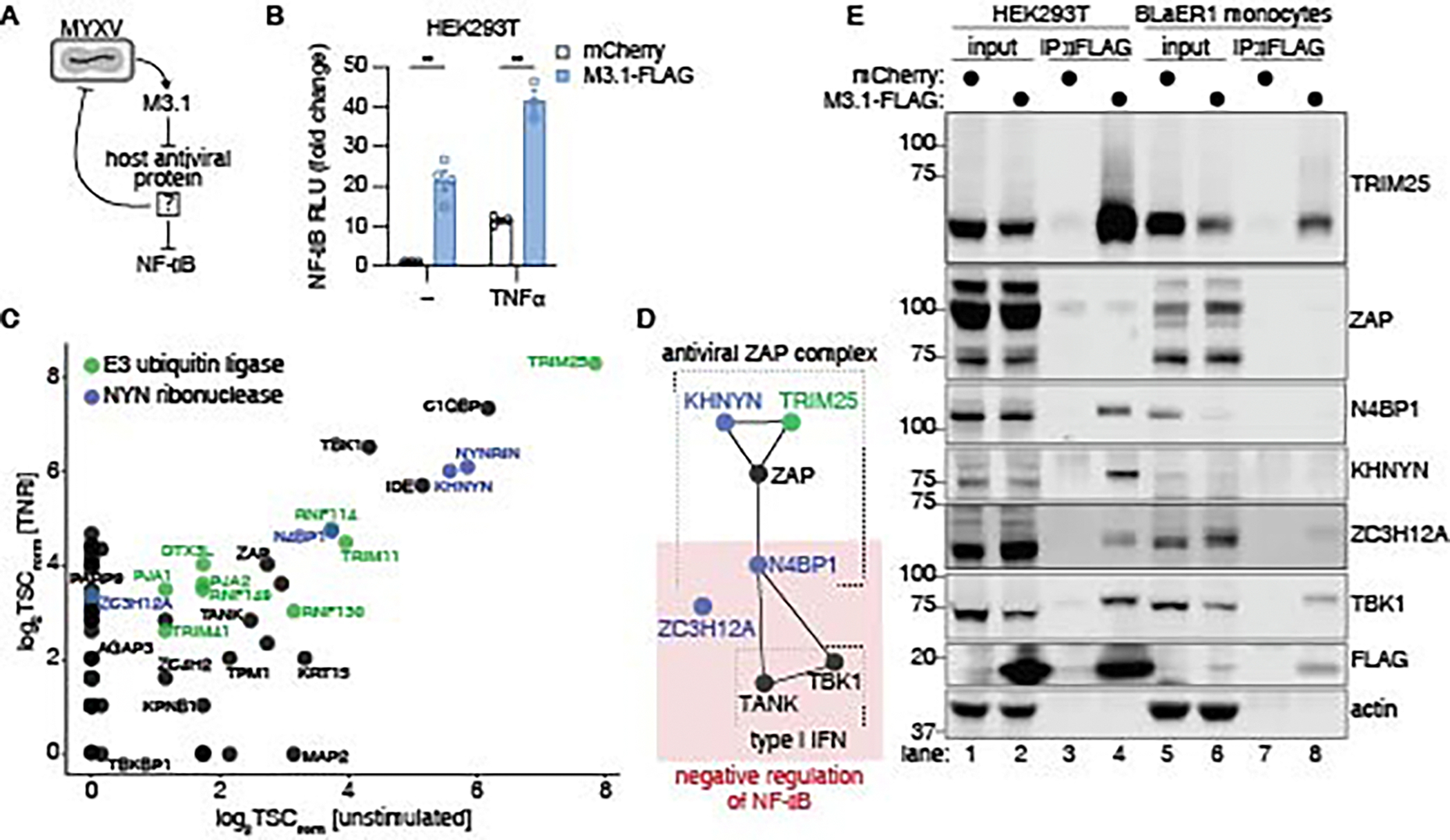
M3.1 interacts with host antiviral factors and NF-κB inhibitors. (**A**) Hypothesized mechanism in which M3.1 inhibits an antiviral host factor that also functions as a negative regulator of NF-κB. (**B**) A luciferase reporter was used to measure NF-κB responses in HEK293T cells transfected with M3.1-FLAG and/or stimulated with TNFα (1 ng/ml). Fold change is relative to unstimulated cells transfected with mCherry. Each data point represents an independent experiment; bars indicate mean ± SEM of 3–4 independent experiments. (**C**) M3.1-FLAG was immunoprecipitated from HEK293T cells that were either unstimulated or treated with TNFα (10 ng/ml) for 8 hours. M3.1 binding partners were identified by mass spectrometry followed by CompPASS analysis. The normalized total spectral count (TSC) is plotted for each M3.1 binding partner. (**D**) String interaction network representing biochemical and/or genetic interactions between M3.1-binding proteins. (**E**) M3.1-FLAG was immunoprecipitated from HEK293T cells and BLaER1 monocytes. Co-purifying proteins were detected by immunoblotting. Images are representative of two independent experiments. ** *P* < 0.01, tested by unpaired t-test with Welch’s correction.

NF-κB signaling is an established host defense response against poxviruses (18). We hypothesized that M3.1 did not evolve to activate NF-κB but to block a different antiviral pathway that inhibits NF-κB as a secondary function. In this scenario, activation of NF-κΒ is an “inadvertent” consequence of M3.1 inhibiting the antiviral pathway (Fig. 2A).

M3.1 is an uncharacterized member of a family of poxvirus Bcl2-like virulence factors that directly bind and antagonize various host antiviral proteins (19, 20). We sought to identify M3.1-interacting proteins in human cells. We expressed M3.1-FLAG in HEK293T cells and confirmed it was able to activate NF-κB responses and enhance TNFα-induced NF-κB responses (Fig. 2B). We then expressed M3.1-FLAG in unstimulated and TNFα-stimulated HEK293T cells and used IP-MS to identify M3.1-interacting proteins. We identified more than 60 M3.1-interacting proteins (z-score > 5), most of which were seen in both the unstimulated and TNFα-stimulated conditions (Fig. 2C) (Data S2).

Several of the M3.1-interacting proteins we identified were known to have antiviral activities. For example, zinc finger antiviral protein (ZAP) restricts a broad range of RNA and DNA viruses, in some cases by binding CpG-rich viral RNA and targeting it for degradation (21–23). Several ZAP cofactors have been identified, including the E3 ubiquitin ligase TRIM25 (24, 25) and the NYN ribonucleases KHNYN and N4BP1 (26–28), all of which were also identified to interact with M3.1 by IP-MS (Fig. 2C).

Moreover, additional NYN ribonucleases, including the KHNYN and N4BP1 paralog NYNRIN, as well as ZC3H12A (MCPIP1, REGNASE-1), also interacted with M3.1. Notably, N4BP1 and ZC3H12A are known negative regulators of NF-κB signaling (29–31). The IP-MS results also indicated that M3.1 interacted with TBK1 and its adaptor TANK. TBK1 is primarily known for its role in inducing type I IFN, a critical host antiviral transcriptional response. Recently, however, suppression of NF-κB by N4BP1 has been shown to involve TBK1 and TANK (32). Thus, many of the proteins we observed to interact with M3.1 are functionally linked to each other and to key antiviral signaling pathways (Fig. 2D).

We used coimmunoprecipitation to validate M3.1 interactions with TRIM25, NYN ribonucleases, and TBK1, in HEK293T cells and BLaER1 monocytes (Fig. 2E). We observed interactions between M3.1 and the ZAP cofactors TRIM25, KHNYN, and N4BP1 in HEK293T cells. However, we were unable to detect a robust interaction between M3.1 and ZAP itself, suggesting a weak or indirect interaction. We also observed that in cells expressing M3.1, the total abundance of some interacting proteins, particularly N4BP1, was decreased (Fig. 2E), raising the possibility that M3.1 promoted degradation of its binding partners. We also noted that some M3.1-interacting proteins migrated at a higher molecular weight in the immunoprecipitated samples compared to input (Fig. 2E), suggesting that these proteins may be post-translationally modified in the presence of M3.1. We concluded that M3.1 interacts with both antiviral proteins and negative regulators of NF-κB in human cells.

### MYXV employs M3.1 to target antiviral ZAP and TBK1 activities

We hypothesized that M3.1 evolved to block the antiviral ZAP complex and/or TBK1-mediated type I IFN signaling, but may trigger a host NF-κB response through suppression of the NF-κB inhibitors N4BP1 and ZC3H12A. While ZAP restricts vaccinia virus (VACV) (33), a poxvirus related to MYXV, it is unknown whether ZAP restricts MYXV.

**Fig. 3.**
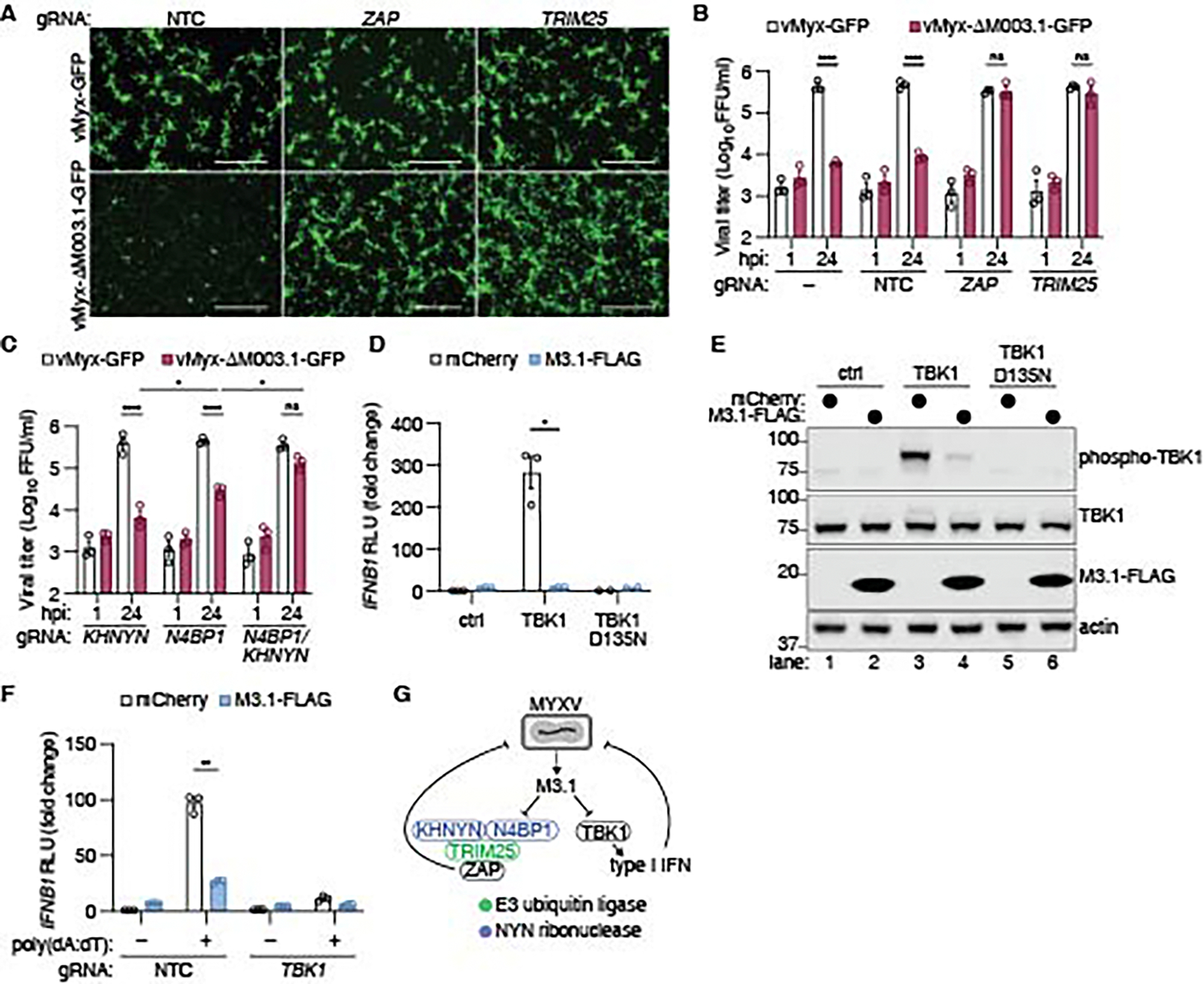
M3.1 blocks ZAP complex antiviral activity and inhibits TBK1-mediated type I IFN induction. (**A**) HEK293T cells nucleofected with Cas9 and two guides targeting the indicated gene or a non-targeting control (NTC) were infected with GFP-expressing MYXV (MOI = 1) for 24 hours. Images were taken using an inverted microscope at 4x magnification and are representative of three independent experiments. Scale bars are 500 μm. (**B-C**) MYXV replication in HEK293T cells infected with an MOI of 1. Viral progeny were quantified at the indicated time points. Each data point represents an independent experiment; bars indicate mean ± SEM 3 independent experiments. (**D**) A luciferase reporter was used to measure *IFNB1* induction in HEK293T cells co-transfected with wild-type or kinase-dead (D135N) TBK1 (25 ng) and M3.1-FLAG (50 ng). Fold change is relative to cells transfected with mCherry. Each data point represents an independent experiment; bars indicate mean ± SEM of 2–3 independent experiments. (**E**) Lysates from (D) were immunoblotted with antibodies against phospho-TBK1, FLAG, and actin. Blots were stripped and re-probed for TBK1. Images are representative of three independent experiments. (**F**) A luciferase reporter was used to measure *IFNB1* induction in HEK293T cells transfected with mCherry or M3.1-FLAG (50 ng) and stimulated with poly(dA:dT) (1 μg/ml) for 24 hours. Fold change is relative to unstimulated cells transfected with mCherry. Each data point represents an independent experiment; bars indicate mean ± SEM of 3 independent experiments. (**G**) Model in which M3.1 inhibits two key antiviral pathways: the ZAP complex and TBK1-mediated type I IFN signaling. * *P* < 0.05; ** *P* < 0.01; **** *P* < 0.0001; ns = not significant, tested by 3-way ANOVA of log-normalized data with Tukey’s post-hoc test (B-C) or by unpaired t-test with Welch’s correction (D, F).

We generated M3.1-deficient MYXV (vMyx-ΔM003.1-GFP) and infected both HEK293T cells and BLaER1 monocytes and found MYXV replicated poorly in BLaER1 monocytes (Fig. S3). Although wild-type MYXV replicated efficiently in HEK293T cells, vMyx-ΔM003.1-GFP replication was severely attenuated (Fig. 3A-B). Genetic deletion of *ZAP*, or its cofactor *TRIM25*, in HEK293T cells by Cas9 ribonucleoprotein (RNP) nucleofection (34) (Fig. S4) rescued vMyx-ΔM003.1-GFP replication, indicating that M3.1 was required to block ZAP antiviral activity during MYXV infection (Fig. 3A-B). While the NYN ribonuclease KHNYN is required for ZAP-mediated restriction of retroviruses, KHNYN is dispensable for ZAP antiviral activity in other contexts (21). Deletion of *KHNYN* failed to rescue vMyx-ΔM003.1-GFP replication, indicating that KHNYN was not required for ZAP-mediated restriction of MYXV in HEK293T cells (Fig. 3C). However, recent work has shown that KHNYN and its paralog N4BP1 have partially redundant functions in the ZAP complex (26). We found that *N4BP1* deletion alone modestly increased vMyx-ΔM003.1-GFP replication, and co-deletion of *N4BP1* and *KHNYN* largely rescued vMyx-ΔM003.1-GFP replication (Fig. 3C). Thus, N4BP1 and KHNYN redundantly promoted ZAP-mediated restriction of MYXV, and MYXV employed M3.1 to escape this restriction.

While our results suggested that a major function of M3.1 was to block ZAP antiviral activity, we considered the possibility that M3.1 may have additional virulence activities. Since we observed an interaction between M3.1 and TBK1, we also tested whether M3.1 affected type I IFN signaling. Overexpression of TBK1 in HEK293T cells activated an *IFNB1* promoter luciferase reporter in a kinase-dependent manner (35, 36) (Fig. 3D). Co-expression of M3.1 blocked TBK1-induced *IFNB1* reporter activation (Fig. 3D) and suppressed TBK1 phosphorylation (Fig. 3E). These data suggested that M3.1 inhibited TBK1-driven type I IFN signaling. M3.1 also blocked *IFNB1* reporter activation in HEK293T cells stimulated with poly(dA:dT), which induces TBK1-dependent type I IFN production in these cells (37, 38) (Fig. 3F). We concluded that M3.1 has two pro-viral functions: inhibition of ZAP and suppression of TBK1-mediated type I IFN induction (Fig. 3G).

### M3.1 triggers NF-κB by blocking N4BP1, ZC3H12A, and TBK1

Since the ZAP complex and type I IFN signaling are important antiviral pathways, we reasoned that hosts may have evolved to guard these pathways, such that their disruption by a virulence factor triggers a compensatory NF-κB response. We hypothesized that the antiviral factors targeted by M3.1 may possess an additional function of repressing NF-κB. Indeed, of the M3.1-interacting proteins we identified, N4BP1, ZC3H12A, and TBK1 are all reported to inhibit NF-κB responses (29–32, 39). We thus predicted that genetic deletion of these M3.1 targets might induce NF-κB signaling, phenocopying M3.1 expression.

**Fig. 4.**
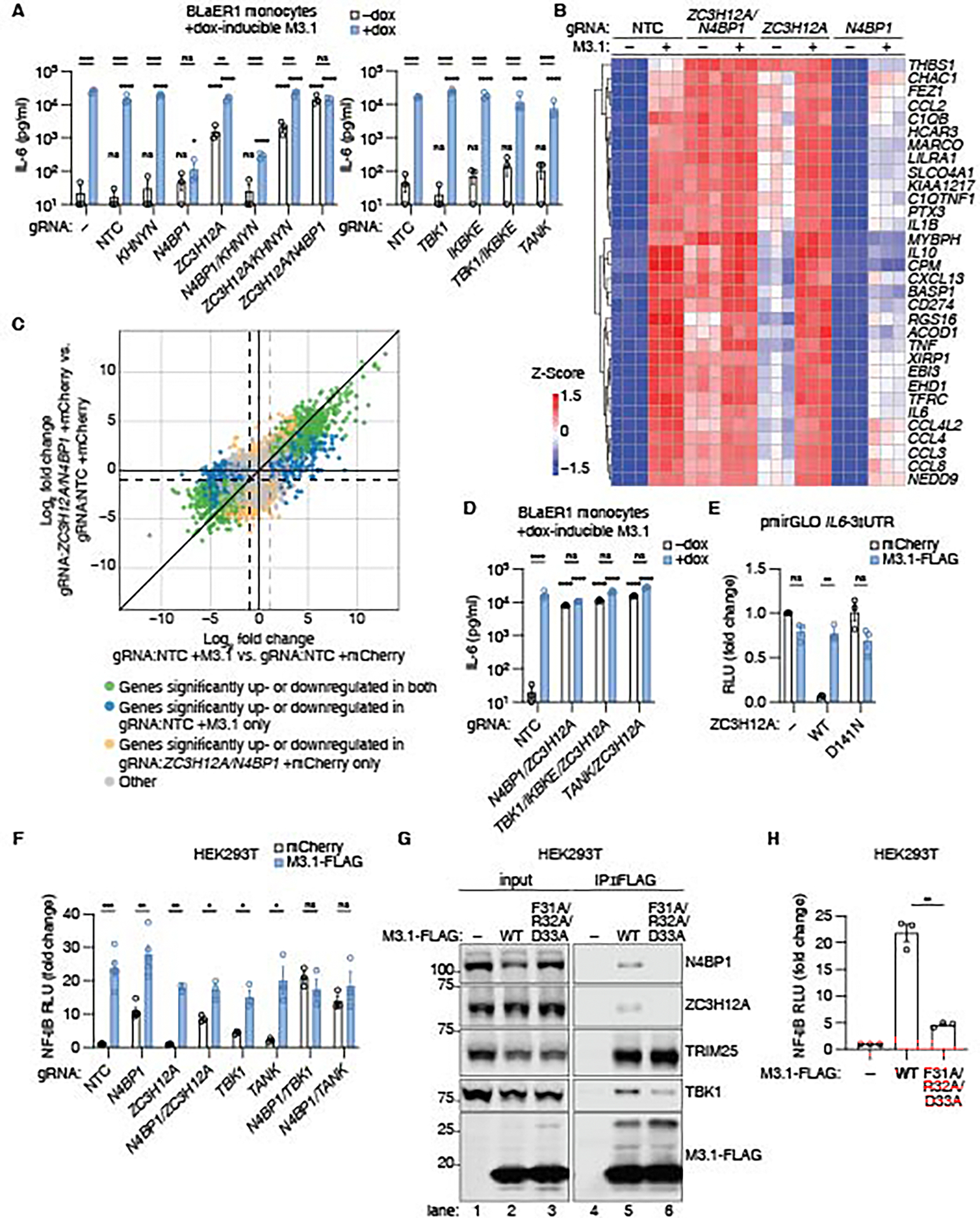
M3.1 unleashes NF-κB signaling by blocking N4BP1, ZC3H12A, and TBK1. (**A, D**) BLaER1 cells expressing doxycycline-inducible M3.1 were nucleofected with Cas9 and two gRNAs targeting the indicated gene or a non-targeting control (NTC). IL-6 secreted by BLaER1 monocytes was measured by ELISA after 24 hours of doxycycline treatment. Each data point represents an independent experiment; bars indicate mean ± SEM of three independent experiments. (**B**) Heatmap analysis of gene expression by BLaER1 monocytes as detected by RNA-seq in three independent experiments. Selected genes are the top 30 genes by variance, as well as known NF-κB targets TNFα and IL-6. (**C**) Plot comparing differential gene expression among BLaER1 monocytes expressing M3.1 and *ZC3H12A/N4BP1*-deficient BLaER1 monocytes expressing mCherry. Dashed lines indicate a log_2_FC cutoff of −1 and 1. (**E**) A pmirGLO dual-luciferase plasmid containing the *IL6* 3′-UTR was used to measure ZC3H12A ribonuclease activity. HEK293T cells were transfected with pmirGLO *IL6*-3′UTR (50 ng), ZC3H12A (25 ng), and M3.1 (50 ng). Luciferase activity was measured after 24 hours. Fold change is relative to mCherry-expressing control cells. Each data point represents an independent experiment; bars indicate mean ± SEM of three independent experiments. (**F**) Cas9-RNP nucleofection was used to disrupt the indicated genes in HEK293T cells, and NF-κB induction by M3.1 was measured using a luciferase reporter. Fold change is relative to mCherry-expressing control cells. Each data point represents an independent experiment; bars indicate mean ± SEM of 3–5 independent experiments. (**G**) FLAG-tagged wild-type (WT) or mutant (F31A/R32A/D33A) M3.1 was immunoprecipitated from HEK293T cells, and binding partners were assessed by immunoblotting. Images are representative of three independent experiments. (**H**) A luciferase reporter was used to measure NF-κB induction in HEK293T cells transfected with M3.1 variants (50 ng). Each data point represents an independent experiment; bars indicate mean ± SEM of three independent experiments. * *P* < 0.05; ** *P* < 0.01; *** *P* < 0.001; **** *P* < 0.0001; ns = not significant, tested by 2-way ANOVA with Šídák’s post-hoc test on log-normalized data (A, D) or unpaired t-test with Welch’s correction (E-F, H). Comparisons were made to untreated control cells unless otherwise indicated (A, D).

We used Cas9-RNP nucleofection (34) to genetically disrupt M3.1-interacting partners in BLaER1 cells that express doxycycline-inducible M3.1 (Fig. S5). We then measured IL-6 production as a readout of NF-κB activation, with and without M3.1 expression (Fig. 4A). In BLaER1 monocytes, only co-deletion of NYN ribonucleases *ZC3H12A* and *N4BP1* was sufficient to phenocopy M3.1 expression, driving IL-6 production that was not further enhanced by M3.1 expression (Fig. 4A).

To characterize the transcriptional profile of *ZC3H12A/N4BP1*-deficient BLaER1 monocytes, we performed RNA-seq of BLaER1 monocytes lacking one or both factors, with and without M3.1 expression. *ZC3H12A/N4BP1*-deficent cells phenocopied M3.1-expressing control cells, and expression of M3.1 had little effect on the transcriptional profile of *ZC3H12A/N4BP1*-deficent cells (Fig. 4B). Moreover, the transcriptional responses of M3.1-expressing cells correlated with those of *ZC3H12A/N4BP1*-deficent cells (Fig. 4C). We concluded that M3.1 unleashed NF-κB signaling in BLaER1 monocytes by inhibiting two negative regulators of NF-κB: ZC3H12A and N4BP1.

The finding that deletion of both *ZC3H12A* and *N4BP1* was necessary to fully recapitulate the effects of M3.1 is consistent with previous reports indicating that mutation of *ZC3H12A* or *N4BP1* alone has modest or negligible effects on NF-κB induction at baseline (29–31, 40). We also observed that the NF-κB response induced by M3.1 was lower in *N4BP1*-deficient cells (Fig. 4A-B). The reason for this was not clear, but it suggested a multilayered interaction between M3.1, N4BP1, and ZC3H12A. Deletion of the related NYN ribonuclease, *KHNYN*, did not affect IL-6 production by M3.1 (Fig. 4A). Likewise, deletion of *ZAP*, *TBK1*, its homolog *IKBKE* (encoding IKKε), or *TANK* did not elicit IL-6 production or affect NF-κB induction by M3.1 (Fig. 4A).

Although N4BP1 and ZC3H12A both contain a NYN ribonuclease domain, they suppress NF-κB responses through distinct mechanisms. NF-κB suppression by N4BP1 is independent of its ribonuclease activity and instead depends on its ubiquitin-binding activity (31, 32). Deletion of *Tbk1* and *Ikbke*, or their adaptor *Tank*, in mouse bone marrow-derived macrophages (BMDMs) phenocopies *N4bp1* deficiency, supporting a model where N4BP1 acts in concert with TBK1/IKKε to limit the duration of IKKα/β signaling (32). Consistent with these data, deletion of *ZC3H12A* in *TBK1/IKBKE*- or *TANK*-deficient BLaER1 monocytes phenocopied *ZC3H12A/N4BP1*-deficient monocytes, and M3.1 expression did not further enhance IL-6 production by these cells (Fig. 4D).

ZC3H12A suppresses NF-κB responses post-transcriptionally by degrading proinflammatory mRNA transcripts via its NYN ribonuclease domain (30). The targets of ZC3H12A, including the *IL-6* mRNA, are recognized via a 3′ UTR stem-loop structure (30). To test if M3.1 blocked ZC3H12A ribonuclease activity, we adapted a luciferase reporter assay where the luciferase mRNA harbors the *IL-6* 3′ UTR (30). Expression of ZC3H12A reduced luciferase activity while expression of the RNase catalytic D141N mutant did not (Fig. 4E). Co-expression of M3.1 prevented ZC3H12A from inhibiting luciferase activity, indicating that M3.1 blocked ZC3H12A ribonuclease activity.

We also investigated whether M3.1 activated NF-κB signaling in HEK293T cells by inhibiting the NYN ribonucleases N4BP1 and ZC3H12A. N4BP1-deficiency alone induced an NF-κB luciferase reporter 10-fold in HEK293T cells, and this was not increased further by co-deletion of *ZC3H12A* (Fig. 4F). Deletion of *ZC3H12A* alone did not affect NF-κB luciferase activity. This finding is consistent with ZC3H12A acting to degrade proinflammatory mRNAs induced by NF-κB, an activity that would not be expected to affect an NF-κB transcriptional reporter.

M3.1 further enhanced NF-κB activity in *N4BP1*-deficient HEK293T cells, indicating that M3.1 had additional NF-κB-enhancing activities in these cells. Because TBK1 is involved in N4BP1-mediated inhibition of NF-κB (32) (Fig. 4D) and is inhibited by M3.1 (Fig. 3D-F), we hypothesized that M3.1 may also promote NF-κB activity via TBK1 inhibition. Deletion of either *TBK1*, or its adaptor *TANK*, in *N4BP1*-deficient HEK293T cells increased NF-κB activation which was not further enhanced by M3.1 (Fig. 4F). We concluded that M3.1 unleashed NF-κB signaling in HEK293T cells by disrupting N4BP1/TBK1-mediated NF-κB suppression.

Our data suggested that M3.1 triggered an NF-κB response by inhibition of NYN ribonucleases. Using a series of N4BP1 domain mutant constructs (Fig. S6A), we observed that the NYN ribonuclease domain was necessary and sufficient for M3.1 to interact with N4BP1 (Fig. S6B). AlphaFold 3 (41) structural modeling predicted that M3.1 interacted with the NYN ribonuclease domains of N4BP1 and ZC3H12A via an interface comprising the F31/R32/D33 residues of M3.1 (Fig. S7). Mutation of these M3.1 residues to alanine ablated the interaction between M3.1 and NYN ribonucleases in HEK293T cells, while largely maintaining interactions with TRIM25 and TBK1 (Fig. 4G). The F31A/R32A/D33A M3.1 mutant exhibited reduced NF-κB activation in both HEK293T cells (Fig. 4H) and BLaER1 monocytes (Fig. S8A-B), indicating that the interaction between M3.1 and N4BP1/ZC3H12A was required for NF-κB induction by M3.1. The F31A/R32A/D33A M3.1 mutant, which retains the ability to interact with ZAP cofactor TRIM25, largely rescued replication of vMyx-ΔM003.1-GFP (Fig. S8C), suggesting that the interaction with TRIM25 may be sufficient for M3.1 to block ZAP antiviral activity.

### TRIM25 promotes M3.1-mediated NF-κB signaling

In addition to interacting with NYN ribonucleases and TBK1, M3.1 also interacted with TRIM25, an E3 ubiquitin ligase and ZAP cofactor. While we did not observe a role for ZAP in M3.1-mediated NF-κB signaling, TRIM25 deficiency reduced NF-κB induction by M3.1 in both BLaER1 monocytes and HEK293T cells (Fig. S9A-B). Overexpression of TRIM25 in HEK293T cells further enhanced NF-κB induction by M3.1 (Fig. S9C), suggesting that TRIM25 promoted M3.1-mediated NF-κB signaling. We hypothesized that TRIM25 might act as a scaffold to promote the stability of M3.1 complexes or, alternatively, that TRIM25 ubiquitin ligase activity might induce NF-κB. We generated two TRIM25 mutants with disrupted ubiquitin ligase activity: C50S/C53S, which blocks zinc finger coordination, and R54P, which prevents the interaction with E2 conjugating enzymes (25, 42). The interaction with M3.1 was reduced by the R54P mutation in TRIM25 and completely blocked by the C50S/C53S mutation (Fig. S9D), suggesting that M3.1 interacted with the TRIM25 RING domain, a model supported by the AlphaFold 3-predicted structure of the complex (Fig. S9E). Despite its reduced interaction with M3.1, the TRIM25 R54P mutant could rescue NF-κB responses in TRIM25-deficent HEK293T cells (Fig. S9C). The TRIM25 C50S/C53S mutant, which does not interact with M3.1, was unable to rescue NF-κB induction. Together, these data support a model where the interaction between M3.1 and TRIM25 promotes M3.1-mediated NF-κB responses, independent of TRIM25 ubiquitin ligase activity. One possible model is that TRIM25 performs a necessary scaffolding function that promotes the activity of M3.1 to disrupt the function of its other target proteins.

### M3.1 promotes degradation of N4BP1

**Fig. 5.**
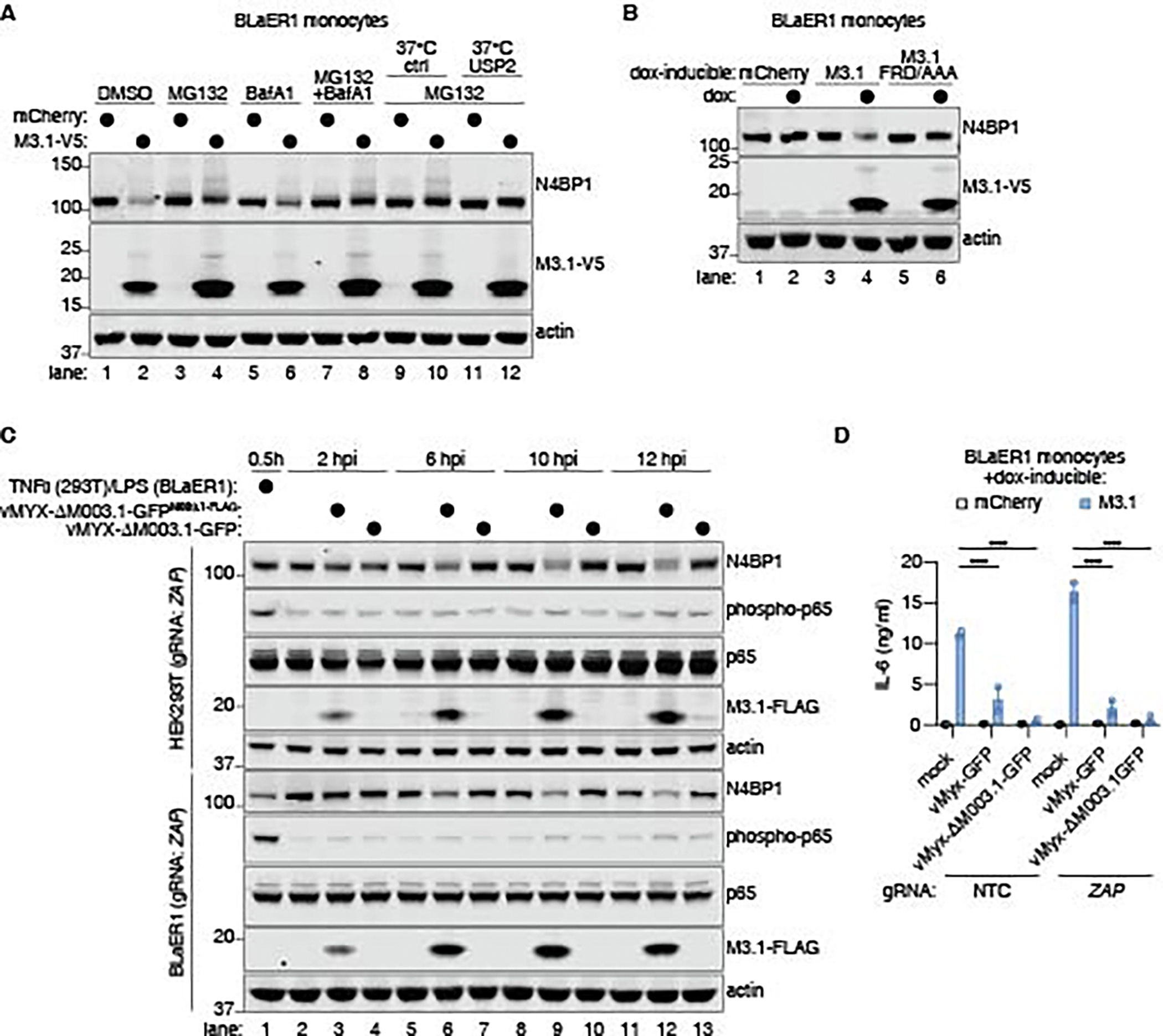
M3.1 promotes degradation of N4BP1. (**A**) BLaER1 monocytes expressing doxycycline-inducible mCherry or M3.1 were treated with doxycycline and MG132 (5 μM) and/or bafilomycin A (125 μM) for 16 hours. Lysates were incubated with or without the deubiquitinase USP2 for 45 minutes at 37°C and immunoblotted as indicated. Images are representative of three independent experiments. (**B**) BLaER1 monocytes were treated with doxycycline for 16 hours and immunoblotted as indicated. Images are representative of two independent experiments. (**C**) ZAP-deficient HEK293T cells and BLaER1 monocytes were infected with the indicated strains of MYXV at an MOI of 3. As a positive control for p65 phosphorylation, HEK293T cells were stimulated with TNFα (100 ng/ml), and BLaER1 monocytes were stimulated with LPS (200 ng/ml). Images are representative of three independent experiments. (**D**) BLaER1 monocytes expressing doxycycline-inducible mCherry or M3.1 were treated with doxycycline and infected with MYXV (MOI = 3) for 24 hours, and secreted IL-6 was measured by ELISA. Each data point represents an independent experiment; bars indicate mean ± SEM of 2 independent experiments. **** *P* < 0.0001, tested by 2-way ANOVA with Šídák’s post-hoc test (D).

We investigated the mechanism by which M3.1 inhibited its targets. Since we observed reduced protein levels of M3.1-interacting proteins, particularly N4BP1 (Fig. 2E), we hypothesized that M3.1 may promote degradation of N4BP1. We treated M3.1-expressing BLaER1 monocytes with MG132, a proteasome inhibitor, and bafilomycin A1, an inhibitor of lysosomal-mediated degradation. We observed that proteasome inhibition enhanced N4BP1 protein levels in M3.1-expressing cells (Fig. 5A). In M3.1-expressing cells treated with MG132, N4BP1 exhibited higher molecular weight bands which would be consistent with ubiquitylation of the protein. Treating lysates with the deubiquitinase USP2 eliminated these higher molecular weight bands, suggesting that N4BP1 was ubiquitylated in the presence of M3.1 (Fig. 5A). Expression of the M3.1 mutant (F31A/R32A/D33A), which cannot interact with NYN ribonucleases, failed to induce N4BP1 degradation (Fig. 5B). Together, these findings suggested that M3.1 promoted the ubiquitylation and proteasomal degradation of N4BP1. N4BP1 is cleaved by caspase-8 downstream of TNFR1, TLR3, and TLR4 (29, 31). Thus, we tested if caspase-8 was required for N4BP1 degradation by M3.1. In cells treated with Z-IETD-FMK, a caspase-8 inhibitor, we still observed M3.1-dependent degradation of N4BP1 (Fig. S10A-C). We concluded M3.1 promoted proteasome-mediated degradation of N4BP1 independent of caspase-8.

We investigated if M3.1 promoted degradation of N4BP1 during MYXV infection. To track M3.1 expression during infection, we complemented vMyx-ΔM003.1-GFP with FLAG-tagged M3.1, which restored virus replication (Fig. S10D). We infected BLaER1 monocytes and HEK293T cells with vMyx-ΔM003.1-GFP or the complemented strain (vMyx-ΔM003.1-GFP^M003.1-FLAG^), using ZAP-deficient cells to eliminate differences in viral replication across strains. M3.1 was expressed within the first two hours of infection (Fig. 5C). By 6 hours post infection, we observed M3.1-dependent loss of N4BP1 protein (Fig. 5C), suggesting that M3.1 promoted N4BP1 degradation during MYXV infection.

To test if M3.1 activated NF-κB signaling during MYXV infection, we monitored phosphorylation of NF-κB p65 in infected cells. MYXV encodes multiple inhibitors of NF-κB (43–45), and we did not detect p65 phosphorylation (Fig. 5C) in either HEK293T cells or BLaER1 monocytes, nor IL-6 induction (Fig. 5D) in BLaER1 cells, during infection. NF-κB activation induced by exogenously expressed M3.1 was also blocked during MYXV infection (Fig. 5D). Thus, although M3.1 promoted degradation of N4BP1 during MYXV infection, we concluded that NF-κB activation was blocked by other virulence factors. Multiple layers of host defenses and poxvirus counterattacks is reminiscent of the “zig-zag” model proposed to underlie the evolution of ETI in plants (3).

### M3.1 homologs modulate NF-κB signaling

**Fig. 6.**
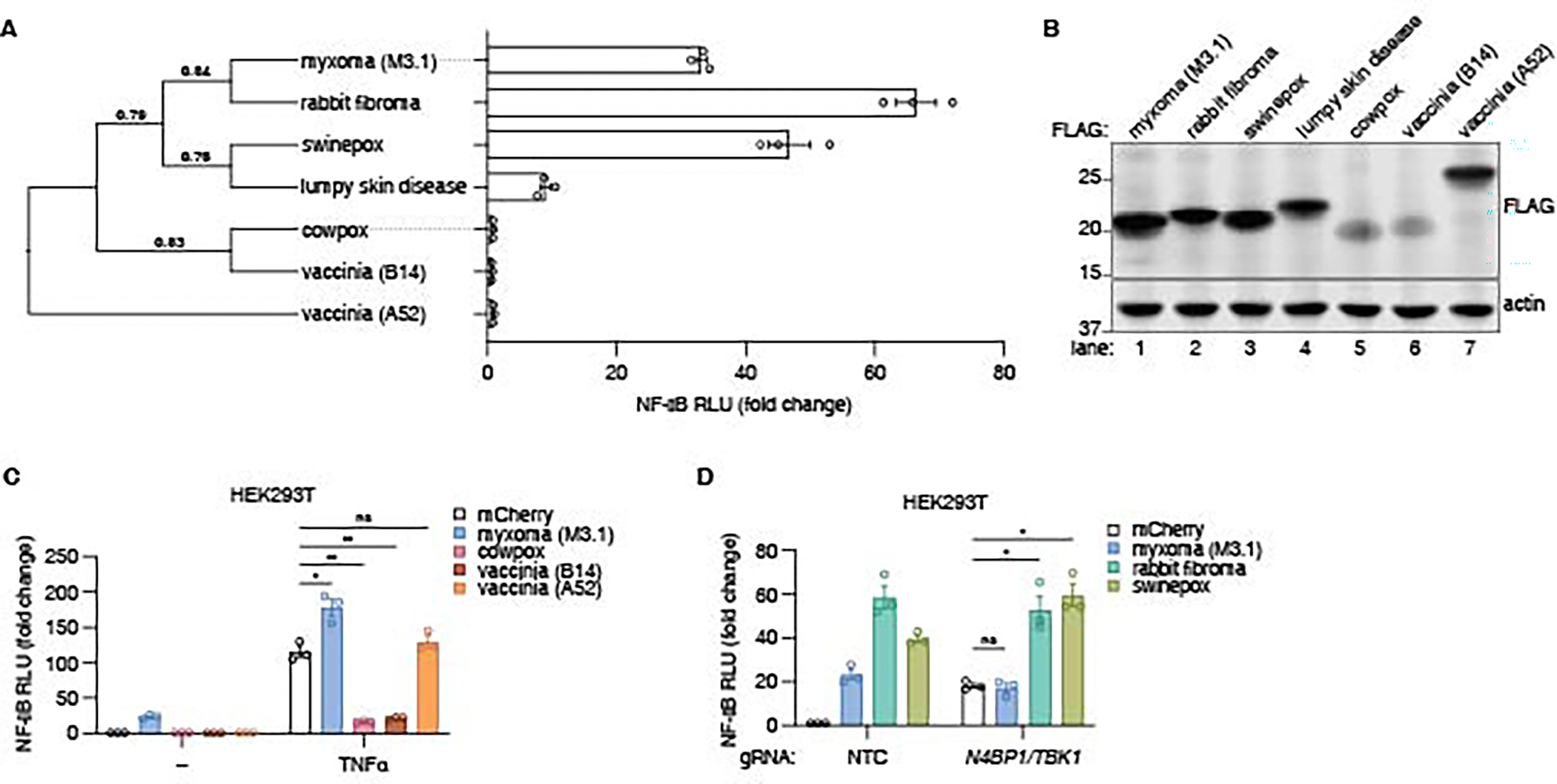
Poxvirus virulence factors modulate NF-κB signaling. (**A**) A phylogeny was built using homologous M3.1 protein sequences from poxviruses. NF-κB induction in HEK293T cells was measured for each homolog (50 ng) using a luciferase reporter. Fold change is relative to mCherry-expressing control cells. Each data point represents an independent experiment; bars indicate mean ± SEM of three independent experiments. (**B**) Lysates in (A) were immunoblotted as indicated. Images are representative of three independent experiments. (**C**) A luciferase reporter was used to measure NF-κB responses in HEK293T cells transfected with M3.1 and its homologs, with and without TNFα (100 ng/ml). Fold change is relative to unstimulated cells transfected with mCherry. Each data point represents an independent experiment; bars indicate mean ± SEM of 3 independent experiments. (**D**) Cas9-RNP nucleofection was used to disrupt the indicated genes in HEK293T cells, and NF-κB induction by M3.1 and its homologs (50 ng) was measured using a luciferase reporter. Fold change is relative to mCherry-expressing control cells. Each data point represents an independent experiment; bars indicate mean ± SEM of three independent experiments. * *P* < 0.05; ** *P* < 0.01; ns = not significant, tested by unpaired t-test with Welch’s correction.

We tested if M3.1 homologs from other poxviruses induced NF-κB signaling. M3.1 homologs from closely related viruses (rabbit fibroma, swinepox, and lumpy skin disease) activated an NF-κB luciferase reporter to varying degrees when expressed in HEK293T cells (Fig. 6A-B). In contrast, M3.1 homologs from more distantly related poxviruses (cowpox, VACV) failed to induce NF-κB (Fig. 6A).

B14, the VACV (Western Reserve strain) homolog of M3.1, has been reported to suppress NF-κB responses by directly binding and inhibiting IKKβ (46). This finding is interesting considering our results that M3.1 interacted with and inhibited TBK1 (Fig. 2–3), a homolog of IKKβ. VACV B14, as well as its cowpox virus homolog, blocked NF-κB induction by TNFα in HEK293T cells (Fig. 6C). VACV A52, a Bcl-2-like virulence factor reported to activate MAPK signaling (47), failed to induce or suppress NF-κB signaling (Fig. 6A-C).

We tested if M3.1 homologs from rabbit fibroma virus and swinepox virus activated NF-κB through a similar mechanism to M3.1. The rabbit fibroma virus and swinepox virus homologs further elevated NF-κB induction in *N4BP1/TBK1*-deficient HEK293T cells, suggesting that these homologs have different and/or additional activities compared to M3.1 (Fig. 6D). These results highlight that the Bcl-2 family of poxvirus virulence factors have evolved to have different functions.

## Discussion

Based on our results, we propose a mechanism of effector-triggered immunity by which human cells can detect poxvirus infection. In our study, a pathway was triggered by M3.1, a virulence factor, or “effector” protein, produced by MYXV. M3.1 is required for MYXV virulence because it disables primary antiviral host factors, yet the antiviral factors attacked by M3.1 inhibited NF-κB responses as their secondary function. We propose, therefore, that host cells protect or “self-guard” (16) their antiviral factors by endowing them with secondary immunoregulatory functions. Since NF-κB promotes anti-poxvirus immunity (18), a potential evolutionary dilemma for poxviruses is that they may either leave primary antiviral immunity intact, or, if they disable it (e.g., with M3.1), unleash a secondary antiviral NF-κB response. We speculate that dual-function “self-guarded” ETI proteins may be a key and underappreciated feature of vertebrate immunity.

The primary antiviral factors targeted by M3.1 were the ZAP complex and TBK1, a kinase that plays a key role in induction of antiviral type I IFN responses (35). One component of the ZAP complex targeted for degradation by M3.1 was N4BP1, a NYN-domain-containing ribonuclease that is thought to provide ZAP with an enzymatic ability to degrade viral RNAs (26, 28). Confusingly, N4BP1 also exhibits a second function in which it negatively regulates NF-κB responses (29, 31). Our results reconcile these two seemingly unrelated functions for N4BP1 in a model where the secondary immunoregulatory function of N4BP1 protects its primary antiviral function: any virus (e.g., MYXV) that attacks N4BP1 may incur the cost of eliciting an antiviral NF-κB ETI response.

Aside from inhibition of N4BP1, inhibition of ZC3H12A and TBK1 also contributed to M3.1-induced NF-κB signaling. In addition to degrading proinflammatory host mRNAs, ZC3H12A can degrade viral RNA (48–50). Thus, the ZC3H12A NYN ribonuclease may also be a self-guarded dual antiviral and immunoregulatory protein. In our hands, however, ZC3H12A deletion did not affect MYXV replication (Fig. S11). TBK1, another protein that promotes antiviral responses and negatively regulates NF-κB signaling, was also blocked by M3.1. We previously identified MORC3 (16) and SP140 (51) as negative regulators of interferon production and as self-guarded antiviral proteins. Thus, host proteins originally identified as negative regulators of immune responses may prove to be a rich source for the discovery of ETI pathways, as any negative regulator could potentially function in pathogen sensing if targeted by a virulence factor.

The ETI pathway described in this study is evolutionarily complex, as highlighted by the diverse functionality we observe in M3.1 homologs from even closely-related poxviruses (Fig. 6). Such diversity is likely driven by the “zig-zag” arms races between poxviruses and their hosts. However, these arms races also make it challenging to experimentally assess the effect of M3.1-induced NF-κB signaling on MYXV replication. During infection, NF-κB induction by M3.1 is suppressed (Fig. 5), likely by other virulence factors known to target the NF-κB signaling pathway (43–45). Evolution of viral mechanisms to block NF-κB induction is consistent with an antiviral role for NF-κB during poxvirus infection (18), but directly testing whether M3.1-induced NF-κB is antiviral would require generating a MYXV strain deficient for all NF-κB inhibitors. We predict that such a virus might activate NF-κB signaling and exhibit attenuated replication, possibly in an M3.1-dependent manner. However, multiple innate immune pathways induce NF-κB (52), which could obscure the effects of M3.1. An advantage of our screening strategy is that by expressing individual virulence factors, we can uncover ETI pathways that are redundant with PTI pathways, or suppressed by other virulence factors during infection.

Our work establishes an experimental screening approach for the discovery of ETI pathways in mammals. We anticipate that this approach can be extended to screen additional virulence factors encoded by both viral and bacterial pathogens and will likely provide further insight into the extent and diversity of mammalian ETI.

## Materials and Methods

### Cell culture

BLaER1 cells were cultured in RPMI 1640 medium (Gibco) supplemented with L-glutamine, sodium pyruvate, 100 U/ml penicillin-streptomycin, and 10% (v/v) FBS (Gibco). HEK293T and RK13 cells were cultured in DMEM medium (Gibco) containing the same supplements. BLaER1 cells were transdifferentiated into monocytes for 5–6 days in medium containing 10 ng/ml of human recombinant (hr) IL-3, 10 ng/ml hr-CSF-1 (M-CSF) (both PeproTech), and 100 nM β-Estradiol (Sigma-Aldrich) as previously described (53). A total of 1.4 million BLaER1 cells were transdifferentiated per well of a 6-well plate, and 7×10^4^ cells were transdifferentiated per 96 well. BLaER1 cells were a gift from Thomas Graf (CRG, Barcelona, Spain) and Veit Hornung (LMU Munich, Germany). HEK293T and RK13 cells were from the UC Berkeley Cell Culture Facility. All cell lines were routinely tested to be free of mycoplasma contaminations.

### Cell stimulation

Cells were stimulated with the indicated concentrations of LPS-EB Ultrapure from *E. coli* O111:B4 (Invivogen), hrTNFα (PeproTech), or poly(dA:dT) (Invivogen) complexed with LyoVec (Invivogen). For activation of doxycycline-inducible transgene expression, cells were treated with 1 μg/ml doxycycline hyclate (Sigma-Aldrich) for the indicated time. Cells were treated with the indicated concentrations of MG132 (Sigma-Aldrich), bafilomycin A (Selleckchem), Z-IETD-FMK (R&D Systems), and GSK-872 (Tocris).

### Cloning

A doxycycline-inducible lentiviral vector (pLIX_403, Addgene #41395) was used for transgene expression in BLaER1 cells. Gateway cloning was used to insert genes in frame with the vector V5 tag unless otherwise stated. For transient transfection in HEK293T cells, the pGCS vector system (Addgene, Kit #1000000107) was used. The pGCS vector system is based on a pCS2+ backbone that has been modified to be compatible with Gateway cloning and to bear either N- or C- terminal epitope tags. Specifically, pGCS1 (no tag), pGSC-N2 (N-terminal 3xHA tag), pGCS-N3 (N-terminal 3xFLAG tag), and pGCS-C3m (C-terminal 3xFLAG) were used in this study. Gene blocks bearing Gateway attB cloning sites were synthesized by Integrated DNA Technologies (IDT) and cloned into the Gateway entry vector pDONR201 using Gateway BP Clonase II Enzyme mix (Invitrogen) according to the manufacturer’s instructions. Gateway LR Clonase II Enzyme mix (Invitrogen) was then used to clone genes into the appropriate pGCS vector. Gene mutations were introduced using the Q5-Site Directed Mutagenesis Kit (New England Biolabs) according to the manufacturer’s instructions.

### Lentiviral transduction

Lentivirus was produced in HEK293T cells in 6-well plates. A 90% confluent well was transfected with 1.56 μg of lentiviral vector, 1.17 μg of pd8.9 packaging vector, and 0.468 μg pVSVG using 8 μl Lipofectamine 2000 (Invitrogen) according to the manufacturer’s instructions. After 8–16 hours, the medium was replaced with DMEM medium containing 30% (v/v) FCS and incubated for 24 hours. Viral supernatants were then harvested, centrifugated at 1000 g for 10 minutes, and filtered through a 0.45 μm filter. Following transduction, cells were cultured for 48 hours and then selected with puromycin (Sigma-Aldrich).

### MYXV ORF arrayed screen

A MYXV ORF library in Gateway entry vectors was constructed as described (10). Gateway LR Clonase II Enzyme mix (Invitrogen) was used to clone MYXV ORFs, as well as control genes mCherry and adenovirus E4ORF3, into a doxycycline-inducible lentiviral vector (pLIX_403, Addgene #41395). Plasmids were Sanger sequenced by Elim Biopharma to verify sequences. Lentivirus was produced in HEK293T cells in an arrayed format using 6-well plates as described above. Viral supernatants were harvested and centrifuged at 1000 g for 10 minutes in deep-well 96-well plates. In place of filtering the supernatants, a blasticidin-resistant (Cas9-expressing) BLaER1 monoclone was transduced in media containing blasticidin (Invivogen) to prevent growth of any remaining HEK293T cells, which are blasticidin-sensitive. After 48 hours, transduced cells were selected with puromycin (Sigma-Aldrich). Successful transductions were achieved for 117 MYXV ORFs (Data S1). BLaER1 B-cells were transdifferentiated into monocytes in a 96-well plate for 5 days as described above and then treated with 1 μg/ml doxycycline hyclate (Sigma-Aldrich) for 24 hours to induce virulence factor expression. Two biological replicates for each MYXV ORF were collected, and prime-seq (17) was adapted to prepare libraries for RNA-seq as follows. Cells were washed in PBS and then lysed in TRK lysis buffer (Omega Bio-tek). Lysates were incubated with Proteinase K (0.6 mg/ml) and EDTA (1 mM) for 10 minutes at 50°C. To terminate the reaction, samples were incubated for 5 minutes on ice with PMSF (2 mM). Lysates were combined with 30% PEG beads prepared in house (Thermo Sera-Mag Speed Beads #45152105050250 resuspended in 2 M NaCl, 10 mM Tris-HCl pH 8.0, 1 mM EDTA, 0.01% Igepal CA-630, 0.05% sodium azide, 30% PEG 8000) at a 1:2 ratio of lysate to beads. The beads were washed 2x in 80% ethanol and eluted in DEPC-treated water. Samples were treated with DNase I (Thermo) in the presence of RNasin Plus Ribonuclease Inhibitor (Promega). Reverse transcription into cDNA was performed as described (17) in the presence of RNasin Plus Ribonuclease Inhibitor (Promega). During cDNA synthesis, prime-seq uses poly(A) priming, template switching, early barcoding, and UMIs to generate 3′ tagged RNA-seq libraries. Samples from all conditions were then pooled and cleaned up with 30% PEG beads at a ratio of 1:1 lysate to beads. Pooled samples were then treated with Exonuclease I (Thermo). cDNA was then amplified using Terra PCR Direct Polymerase Mix (Takara) for 9 cycles. Libraries were then tagmented (4–5 technical replicates per library) and amplified using Nextera XT DNA Library Preparation Kit (Illumina) according to the manufacturer’s instructions. The following primers were used for library amplification and i7 adaptor addition:

3′ enrichment primer (P5NEXTPT5): AATGATACGGCGACCACCGAGATCTACACTCTTTCCCTACACGACGCTCTTCCGATCT

i7 index primer: CAAGCAGAAGACGGCATACGAGAT[i7]GTCTCGTGGGCTCGG

Libraries were then pooled and sequenced by the Vincent J. Coates Genomics Sequencing Lab (UC Berkeley) on two Illumina NovaSeq 6000 SP flow cells using 10X sequencing specifications. zUMIs (54) was used to demultiplex samples, collapse UMIs, and align reads to human (GRCh38) and myxoma virus (GCF_000843685.1) reference genomes. Differential gene expression analysis was conducted by iterating through each condition and using DESeq2 (1.42.1) to compare gene expression to that of mCherry-expressing control samples.

### RNA-seq

2.8 million BLaER1 monocytes expressing doxycycline-inducible mCherry or M3.1 were treated with 1 μg/ml doxycycline hyclate (Sigma-Aldrich) for 24 hours. Three biological replicates were collected for each genotype and condition. RNA was isolated using the Omega Biotek Total RNA Kit I according to the manufacturer’s instructions. DNA was removed with TURBO DNase (Invitrogen) in the presence of RNasin Plus Ribonuclease Inhibitor (Promega), and RNA was isolated with RNAClean XP beads (Beckman Coulter). For Fig. 1C-D, library preparation and sequencing were performed by the QB3-Berkeley Genomics core using the KAPA mRNA Hyper Prep kit (Roche KK8581). Libraries were sequenced on an Illumina NovaSeq 6000 S4 flow cell (2×150 bp, 25M paired end reads per sample). For Fig. 4B-C, library preparation and sequencing were conducted by Azenta Life Sciences using the NEBNext Ultra II RNA Library Prep Kit for Illumina (New England Biolabs). Libraries were sequenced on an Illumina NovaSeq (2×150 bp, 20M paired end reads per sample). Sequencing quality of fastq files was evaluated with FastQC, and adaptors were trimmed using Cutadapt (1.18). Paired end RNA-seq reads were aligned to the reference genome (GRCh38) using STAR (2.7.1a). Fragment counts were quantified using featureCounts from the Subread (2.0.3) package, and differential gene expression analysis was conducted using DESeq2 (1.42.1). Gene set enrichment analysis was conducted using clusterProfiler (4.10.1). The Molecular Signatures Database (MSigDB) hallmark gene sets were downloaded using the R package msigdbr (7.5.1).

### Cytokine quantification

Cytokine secretion was quantified by ELISA of cell-free supernatants following manufacturer’s instructions (human IL-6: BD 555220).

### Immunoprecipitation mass spectrometry

Ten 15-cm plates per condition were seeded with 4 million HEK293T cells. After 24 hours, cells were transfected with 5 μg M3.1–3xFLAG per plate. 48 hours post transfection, cells were treated with 10 ng/ml hrTNFα (PeproTech) or media control for 8 hours and then harvested by scraping in PBS. Cell pellets were flash frozen in liquid nitrogen. Cells were lysed in lysis buffer (40 mM HEPES pH 7.5, 150 mM NaCl, 0.2% NP-40, cOmplete EDTA-free protease inhibitor cocktail tablets (Sigma Aldrich)) for 60 minutes at 4°C. Lysates were spun at 21,000 g for 30 minutes, and the supernatant was added to 90 μl of prewashed ANTI-FLAG^®^ M2 Affinity Agarose Gel slurry (Sigma-Aldrich, A2220). After 1.5 hours of rocking, the beads were spun down and washed 5x with lysis buffer. Beads were then washed 2x in PBS with 0.2% NP-40. Proteins were eluted with 500 μg/ml 3xFLAG peptide (Millipore, #F4799) in PBS with 0.2% NP-40. Elutions were precipitated in 20% final concentration of trichloroacetic acid on ice overnight. Precipitations were spun at 21,000 g for 10 minutes and washed 3x in ice cold acetone and dried. The pellets were solubilized in 8 M urea 100 mM Tris pH 8.5, treated with TCEP and iodoacetamide, and digested overnight with trypsin (Promega). Samples were analyzed by Multidimensional Protein Identification Technology (MudPIT) at the Vincent J. Coates Proteomics/Mass Spectrometry Laboratory (UC Berkeley). M3.1-interacting proteins were identified by CompPASS analysis (55) by comparing the samples to over 70 similarly performed anti-FLAG immunoprecipitations from HEK293T cells. Total spectral counts were normalized to 1000 bait counts, and results with a z-score greater than 5 were plotted. See Data S2 for CompPASS output.

### Coimmunoprecipitation

Cells were lysed in lysis buffer (40 mM HEPES pH 7.5, 150 mM NaCl, 1% NP-40, Pierce Protease and Phosphatase Inhibitor EDTA-free mini tablets (Thermo)) for 30 minutes on ice. Lysates were clarified by centrifugation for 30 minutes at 4°C, and 5% of clarified lysate was removed as an input. Remaining sample was added to 20 μL of washed ANTI-FLAG^®^ M2 Affinity Agarose Gel slurry (Sigma-Aldrich, A2220) and rotated for 1–2 hours at 4°C. Beads were washed four times and eluted with Laemmli buffer by boiling for 7 minutes. Samples were then analyzed by immunoblot.

### Immunoblotting and antibodies

Whole cell lysates were prepared by lysing cells in RIPA buffer (150 mM NaCl, 5 mM EDTA pH 8.0, 50 mM Tris-HCl pH 8.0, 1% NP-40, 0.5% sodium deoxycholate, 0.1% SDS) supplemented with Pierce Protease and Phosphatase Inhibitor EDTA-free mini tablets (Thermo) for 20 minutes on ice. Lysates were clarified by centrifugation for 20 minutes at 4°C. To remove ubiquitin chains, lysates were incubated with 1 μM recombinant USP2 catalytic domain (R&D Systems) for 45 minutes at 37°C. Laemmli buffer was added to a final concentration of 1x and lysates were boiled at 95°C for 7 minutes. Samples were run on 4–12% Bis-Tris protein gels (Invitrogen) and then transferred to Immobilon-FL PVDF membranes (Millipore Sigma). Membranes were blocked with Li-Cor Odyssey blocking buffer and then incubated with primary antibodies overnight at 4°C. Following incubation with appropriate secondary antibodies, blots were imaged using the Li-Cor Odyssey platform. If indicated in the figure legend, blots were stripped with NewBlot^™^ IR Stripping Buffer (Licor) according to the manufacturer’s instructions and re-probed. The complete gel images are shown in Fig. S12-14. The following antibodies were used: Phospho-NF-κB p65 (Ser536) (93H1) (Cell Signaling, #3033), NF-κB p65 (L8F6) (Cell Signaling, #6956), IκBα (Cell Signaling, #9242), V5-Tag (D3H8Q) (Cell Signaling, #13202), Actin (Santa Cruz, sc-47778), TRIM25 (Abcam, ab167154), ZC3HAV1/ZAP (Proteintech, 16820–1-AP), N4BP1 (Thermo Fisher/Bethyl Laboratories, #A304–628A-T), KHNYN (Santa Cruz, sc-514168), ZC3H12A (GeneTex, GTX110807), TBK1/NAK (D1B4) (Cell Signaling, #3504), Phospho-TBK1/NAK (Ser172) (D52C2) (Cell Signaling, #5483), FLAG M2 (Sigma-Aldrich, F3165), FLAG (D6W5B) (Cell Signaling, #14793), TANK (Cell Signaling, #2141), IKKε (Cell Signaling, #2690), HA-tag (C29F4) (Cell Signaling, #3724), IRDye 800 Donkey anti-Mouse IgG Secondary Antibody (Li-Cor, 926–32212), Alexa Fluor 680 Goat anti-Rabbit IgG Secondary Antibody (Invitrogen, A-21109).

### Gene disruption by Cas9-RNP nucleofection

Polyclonal gene-deficient BLaER1 cells and HEK293T cells were generated as follows. Two gRNAs per gene were designed to target early coding exon(s) of the respective gene using ChopChop (56). BLaER1 cells expressing doxycycline-inducible mCherry or M3.1 or HEK293T cells were harvested and washed in PBS. 2 million cells were nucleofected with Alt-R^®^ S.p. Cas9 Nuclease V3 (IDT) complexed with two gRNAs per gene (Synthego, sgRNA EZ Kit) and Alt-R^®^ Cas9 Electroporation Enhancer (IDT) in Lonza P3 buffer (Lonza, V4XP-3032) as described (34). Nucleofection was performed with a Lonza 4DNucleofector Core Unit (AAF-1002B) using the program CM-137. Negative Control sgRNA (mod) #1 (Synthego) was used as a non-targeting control (NTC). Knockout efficiency was evaluated by immunoblot (Fig. S4-5). Deletion of multiple genes was performed sequentially. The following gRNA sequences were used (PAM is highlighted in bold): GACCTTGCATCAGTAACCGA**AGG** (*N4BP1* gRNA 1), ACAGGCCCTCGATACGGCCG**CGG** (*N4BP1* gRNA 2), ATGGAAACGAGGCGCCCGAG**GGG** (*KHNYN* gRNA 1), GAGCTCCCCCTAGTGACGGC**AGG** (*KHNYN* gRNA 2), GAATCGGCACTTGATCCCAT**AGG** (*ZC3H12A* gRNA 1), GGTCATCGATGGGAGCAACG**TGG (***ZC3H12A* gRNA 2), GTCGTGCCTGAATGAGACGT**GGG** (*TRIM25* gRNA 1), GCGGCGCAACAGGTCGCGAA**CGG** (*TRIM25* gRNA 2), CGGACTGCGAATAGTTGCAC**CGG** (*ZC3HAV1*/ZAP gRNA 1), AAAATCCTGTGCGCCCACGG**GGG** (*ZC3HAV1*/ZAP gRNA 2), GGTAGTCCATAGGCATTAGA**AGG** (*TBK1* gRNA 1), AAATATCATGCGTGTTATAG**GGG** (*TBK1* gRNA 2), TGCATCGCGACATCAAGCCG**GGG** (*IKBKE*/IKKε gRNA 1), GCCCCAGCAAAAAGCGTTCG**GGG** (*IKBKE*/IKKε gRNA 2), GCAGAGAATACGTGAACAAC**AGG** (*TANK* gRNA 1), CCACAAGATAAAGTGATTTC**AGG** (*TANK* gRNA 2).

### MYXV preparation

Recombinant viruses were generated by homologous recombination. The DNA fragment containing a reporter gene that replaced the target ORF and appropriate flanking sequences for the homologous recombination was synthesized and cloned in the pUC57 plasmid by GenScript. To generate the vMyx-ΔM003.1-GFP virus, the M3.1 ORF was replaced with a GFP expression cassette (driven by a poxvirus synthetic early/late promoter) flanked by the M-T2 (partial) and M003.2 sequences. vMyx-ΔM003.1-GFP complemented/revertant virus with FLAG-tagged M3.1 ORF under its native promoter (vMyx-ΔM003.1-GFP^M003.1-FLAG^) was also generated by homologous recombination. RK13 cells were infected with the wild-type MYXV-Lau strain for one hour, and then the unbound virus was removed. Cells were then transfected with the recombination plasmid using Effectene transfection reagent (Qiagen). GFP expression from the recombination plasmid was monitored using a Leica fluorescence microscope. The cells were scraped and collected with media 48 hours post-infection and stored at −80°C until processed. The samples were freeze-thawed at −80°C and 37°C three times and sonicated with a Cup Horn Sonicator for 1 minute to release the viruses from the infected cells. The samples were serially diluted with media, plated on RK13 cells, and infected for 1 hour. Media was removed and layered with 1% low-melting agarose diluted with the media. After 48 hours of infection, fluorescent foci were selected using the microscope, and foci were picked using pipette tips and diluted in media. Subsequently, multiple rounds of foci purification were performed until the pure foci of only recombinant viruses were isolated. The virus was amplified from a single foci in RK13 cells, and the purity of the virus was confirmed by PCR using appropriate primers. The construction of wild-type MYXV that expresses GFP under the control of a poxvirus synthetic early/late promoter (vMyx-GFP) was described previously (57). All the viruses were amplified after infecting RK13 cells grown in multiple T150 cell culture dishes. Viruses were then purified by centrifugation through a sucrose cushion as described previously (58).

### Myxoma virus infections

Transdifferentiated BLaER1 monocytes and HEK293T cells were infected with MYXV at the indicated MOI. For virus replication assays, cells were infected for 1 hour, and then unbound virus was removed and replaced with fresh media. Cells were scraped and collected with media at various times post-infection and stored at −80°C until processed. The samples were freeze-thawed at −80°C and 37°C three times to release the virus from infected cells. The virus was titrated onto confluent RK13 monolayers by serial dilution in triplicate. After 48 hours of infection, fluorescent foci were quantified using a Zeiss Cell Discoverer 7 at the Cancer Research Laboratory (CRL) Molecular Imaging Center (UC Berkeley) and used to calculate the virus titer.

### AlphaFold 3 structure predictions

Amino acid sequences for proteins of interest were obtained from NCBI. The AlphaFold 3 (41) server (https://alphafoldserver.com/) was used to predict the structures of complexes formed between MYXV M3.1 (QCO69335.1) and the following proteins: N4BP1 (NP_694574.3), ZC3H12A (NP_001310479.1), and TRIM25 (NP_005073.2). Output models were visualized in ChimeraX (1.8). Predicted Aligned Error (PAE) plots were visualized in PAE Viewer (59).

### NF-κB and IFNβ luciferase assays

HEK293T cells were plated in white-walled 96-well plates and incubated overnight. Using Lipofectamine 2000 (Invitrogen), HEK293T cells were co-transfected with the indicated constructs, reporter constructs (50 ng) expressing firefly luciferase under control of the *ELAM* promoter (NF-κB reporter) or the *IFNB1* promoter, and a *Renilla* luciferase-expressing construct (10 ng) to control for transfection efficiency. After 24–36 hours, luciferase activity was measured using the Dual-Glo Luciferase Assay System (Promega) according to the manufacturer’s instructions. To account for transfection efficiency, the firefly luciferase values were divided by *Renilla* luciferase values for each well.

### ZC3H12A ribonuclease activity luciferase assay

The pmirGLO Dual-Luciferase expression vector (Promega) was used to measure ZC3H12A ribonuclease activity. A ZC3H12A target, the human *IL-6* 3’UTR, was cloned downstream of the firefly luciferase gene. Using Lipofectamine 2000 (Invitrogen), HEK293T cells were co-transfected with the pmirGLO-IL6 3’UTR construct (50 ng) and constructs encoding ZC3H12A, M3.1, and/or mCherry controls. After 24 hours, luciferase activity was measured using the Dual-Glo Luciferase Assay System (Promega) according to the manufacturer’s instructions. The internal *Renilla* luciferase control was used to normalize for transfection efficiency.

### Phylogenetic analysis

Position-specific iterated (PSI)-BLAST was used to identify M3.1 homologs. Homologs that were able to be expressed in HEK293T cells were selected for further analysis. VACV A52, a Bcl-2-like protein reported to activate MAPK signaling, was chosen as an outgroup (19, 47). The following protein sequences were downloaded from NCBI: myxoma virus M3.1 (QCO69335.1), rabbit fibroma virus (NP_051888.1), lumpy skin disease virus (AYV61288.1), swinepox virus (QQG31492.1), cowpox virus (NP_619989.1), vaccinia virus (Western Reserve strain) B14 (UZL86927.1), vaccinia virus (Western Reserve strain) A52 (YP_233060.1). Above sequences were aligned on Phylogeny.fr with MUSCLE using the default settings. Maximum likelihood phylogenetic trees were generated with PhyML using 100 bootstrap replicates.

### Statistical analysis

Statistical analysis was performed in GraphPad Prism 10. Statistical tests are indicated in the figure legends. Each data point is from an independent experiment and represents the average of 2–3 technical replicates.

## Supplementary Material

Supplementary Figures

Supp data S1

Supp data S2

Figs. S1 to S14

Data S1 and S2

## Data Availability

Code for RNA-seq analysis and figure generation has been deposited on Zenodo (60–62). Raw and processed RNA-seq data has been deposited at NCBI Gene Expression Omnibus with the accession numbers GSE288433, GSE287860 and GSE288000. All other data needed to evaluate the conclusions in the paper are available in the main text or the supplementary materials. Mutant cell lines can be generated using methods detailed in the methods section. Viruses are available upon request without an MTA.
