# Supplementary material for: Poxvirus attack of antiviral defense pathways unleashes an effector-triggered NF-κB response
Source: Science. Author manuscript; Available in PMC 2026 Apr 2. (PMC13041778; doi:10.1126/science.adw4937)
Supplement: Supplementary Figures [file NIHMS2148737-supplement-Supplementary_Figures.pdf]

Supplementary Materials for

Poxvirus attack of antiviral defense pathways unleashes an effector-triggered NF-κB response

Brenna C. Remick, Joshua Q. Mao, Andrew G. Manford, Ami D. Gutierrez-Jensen, Allon Wagner, Michael Rape, Grant McFadden, Masmudur M. Rahman, Moritz M. Gaidt, Russell E. Vance

Corresponding authors: rvance@berkeley.edu, moritz.gaidt@imp.ac.at

The PDF file includes:

Figs. S1 to S14

Other Supplementary Materials for this manuscript include the following:

Data S1 and S2

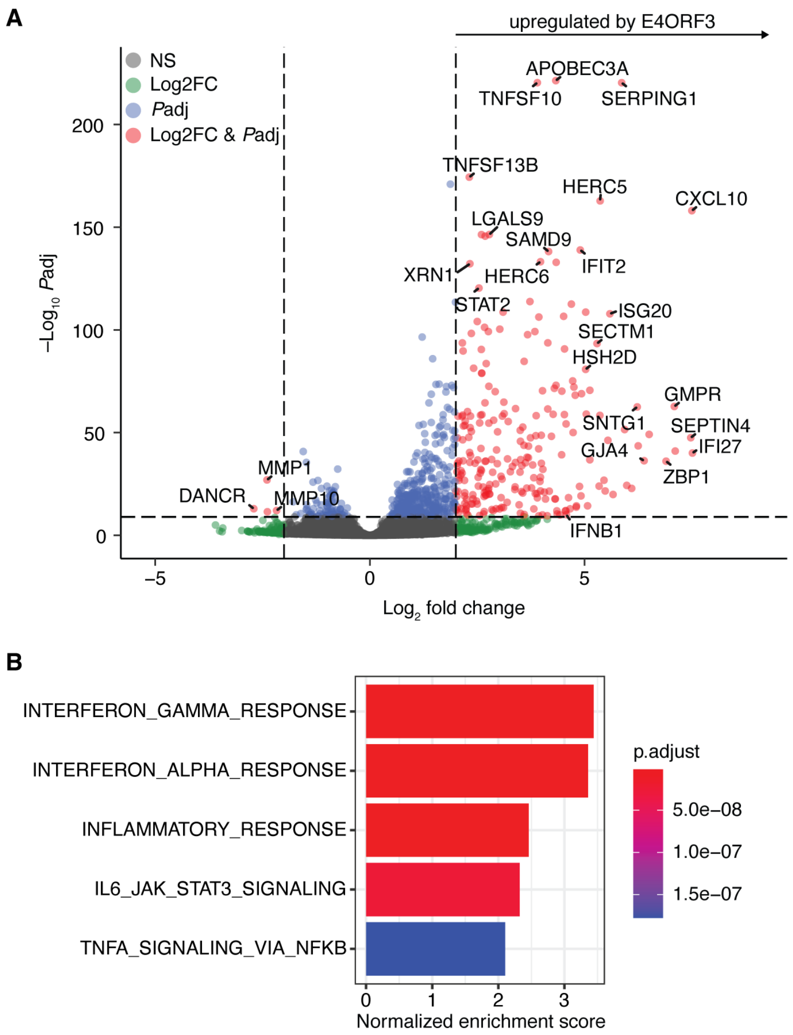

Fig. S1. Adenovirus E4ORF3 elicits a type I IFN response in BLaER1 monocytes. (**A**) Volcano plot from the screen of differentially expressed genes in BLaER1 monocytes expressing E4ORF3 for 24 hours compared to mCherry. Dashed lines indicate a log_2FC cutoff of ±2 and a *P*adj cutoff of 10e-10. (**B**) MSigDB Hallmark gene sets enriched in E4ORF3-expressing BLaER1 monocytes in (A).

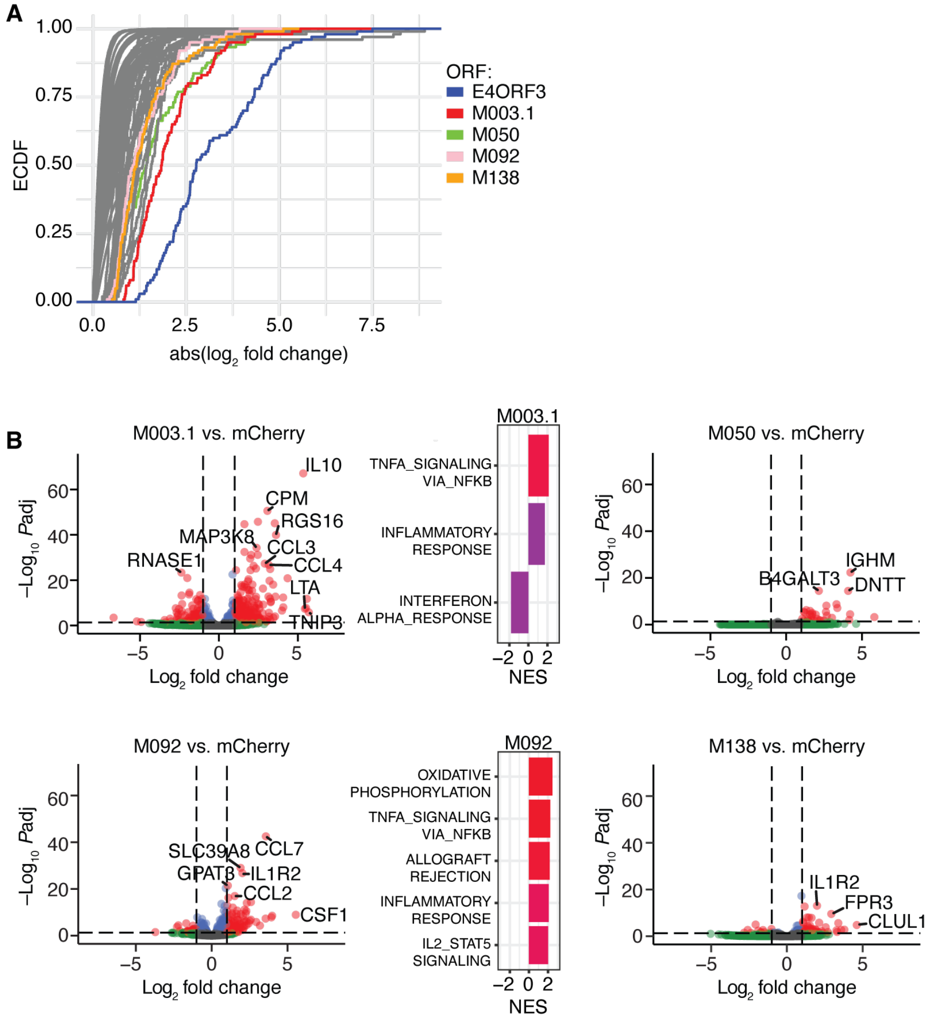

Fig. S2. Arrayed screening of the MYXV ORF library identifies virulence factors that elicit host transcriptional responses. (**A**) For each viral ORF, DESeq2 was used to identify differentially expressed host genes compared to the mCherry control. The empirical cumulative distribution function (ECDF) of the absolute fold change was plotted for the top 100 genes with the lowest *P*adj values for each ORF. Colored lines represent specific MYXV ORFs that exhibit a distinct shift in the ECDF curve, suggesting a notable host transcriptional response induced by these ORFs. (**B**) Volcano plots and MSigDB Hallmark gene set enrichment for BLaER1 monocytes expressing the indicated MYXV ORF. The volcano plot shows differential gene expression relative to mCherry-expressing control samples. Dashed lines indicate a $\log_2$FC cutoff of ±1 and a *P*adj cutoff of 0.05. The gene set enrichment plot displays normalized enrichment scores (NES) for significantly enriched Hallmark gene sets.

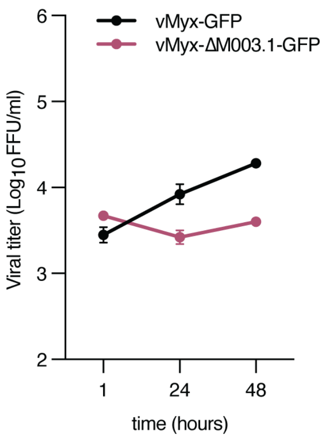

Fig. S3. MYXV replicates poorly in BLaER1 monocytes. BLaER1 monocytes were infected with MYXV with an MOI of 0.1. Viral progeny were quantified at the indicated time points.

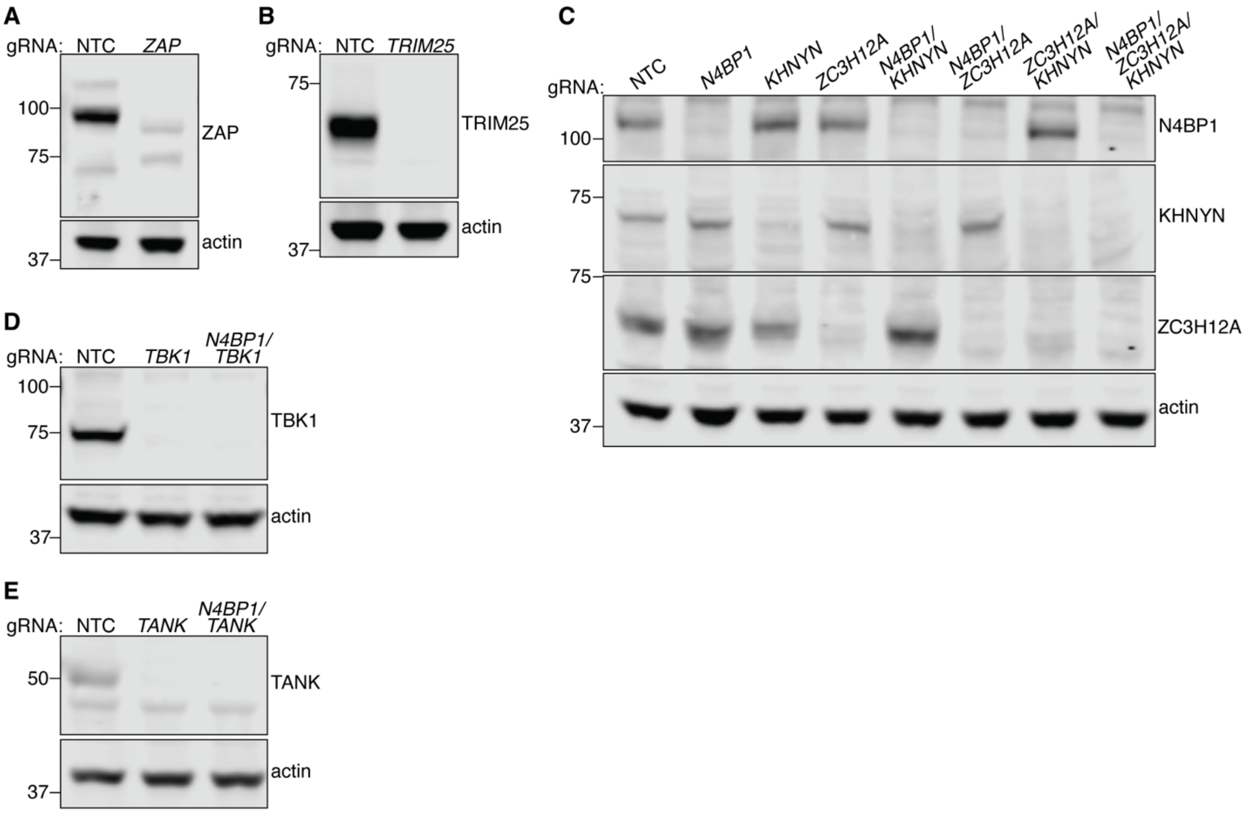

Fig. S4. Gene knockout by Cas9-RNP nucleofection of HEK293T cells. HEK293T cells were nucleofected with Cas9 and two guides per gene. Knockout efficiency in the polyclonal cell population was assessed by immunoblotting for ZAP (**A**), TRIM25 (**B**), NYN ribonucleases N4BP1, KHNYN, and ZC3H12A (**C**), TBK1 (**D**), and TANK (**E**). Double or triple knockouts were generated sequentially.

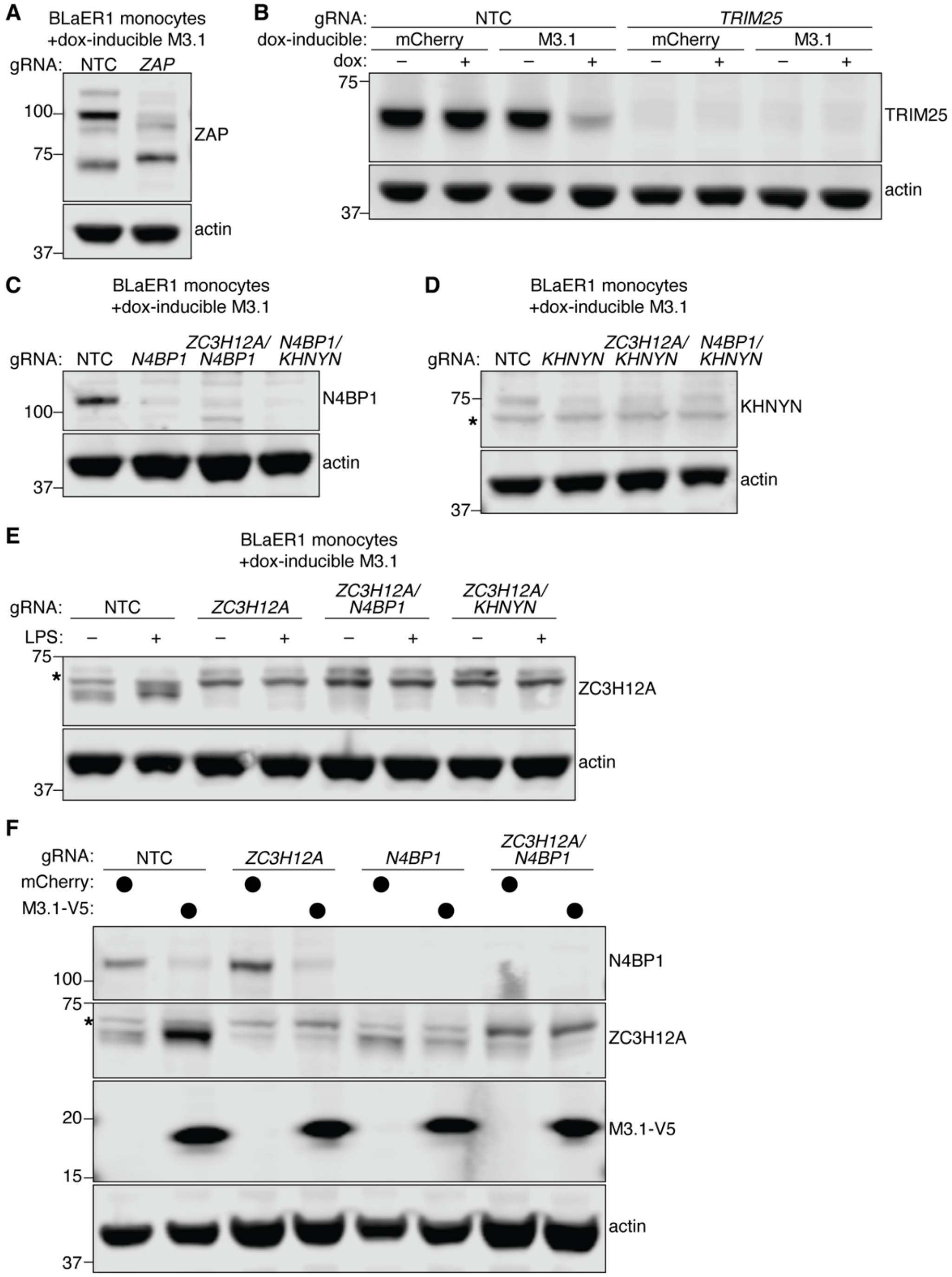

Fig. S5. Gene knockout by Cas9-RNP nucleofection of BLaER1 cells. BLaER1 B-cells expressing doxycycline-inducible mCherry or M3.1 were nucleofected with Cas9 and two guides per gene. B-cells were transdifferentiated for 5 days into monocytes, and then knockout efficiency in the polyclonal cell population was assessed by immunoblotting for ZAP (**A**), TRIM25 (**B**), N4BP1 (**C**), KHNYN (**D**), and ZC3H12A (**E**). Since ZC3H12A is an NF-κB-inducible gene, monocytes were stimulated with 200 ng/ml LPS overnight to boost ZC3H12A expression in (E). (**F**) Knockout efficiency and M3.1 expression were verified for RNA-seq samples treated with doxycycline for 24 hours. Double knockouts were generated sequentially. *Indicates non-specific band.

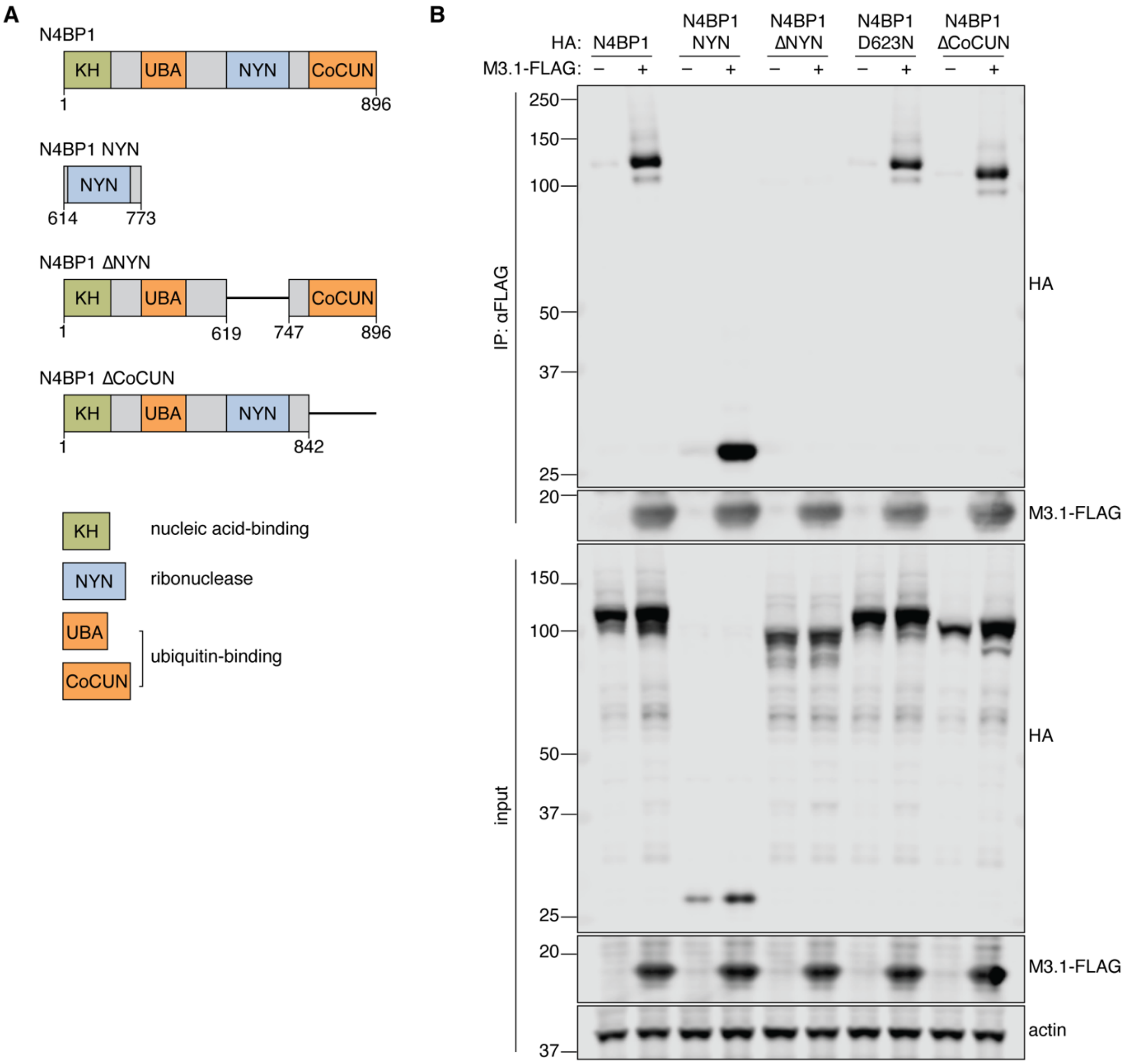

Fig. S6. The NYN ribonuclease domain of N4BP1 is necessary and sufficient to interact with M3.1. (**A**) Schematic of N4BP1 domain mutant constructs. KH: K Homology; UBA: Ubiquitin-associated; NYN: N4BP1, YacP-like Nuclease; CoCUN: Cousin of CUBAN. (**B**) Constructs encoding HA-tagged full length N4BP1, N4BP1 NYN domain only, N4BP1ΔNYN, N4BP1 ribonuclease-dead (D623N), and N4BP1ΔCoCUN were expressed in HEK293T cells with M3.1-FLAG or control. M3.1-FLAG was immunoprecipitated and the interaction with N4BP1 variants was assessed by immunoblotting.

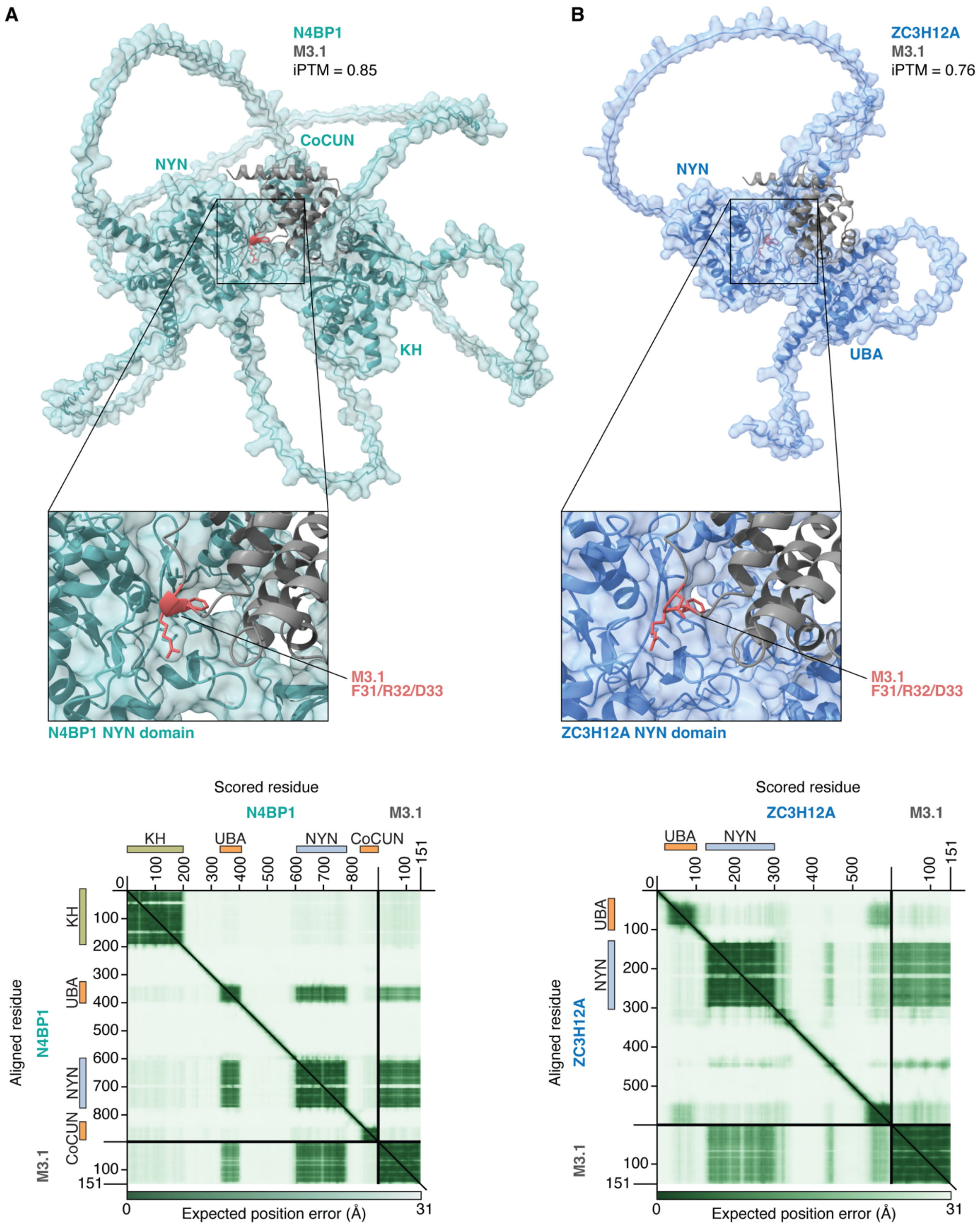

Fig. S7. AlphaFold 3-predicted structures of M3.1 with N4BP1 and ZC3H12A. AlphaFold 3 was used to generate predicted structures of the complex of M3.1 with N4BP1 (**A**) and with ZC3H12A (**B**). Corresponding PAE plots are shown.

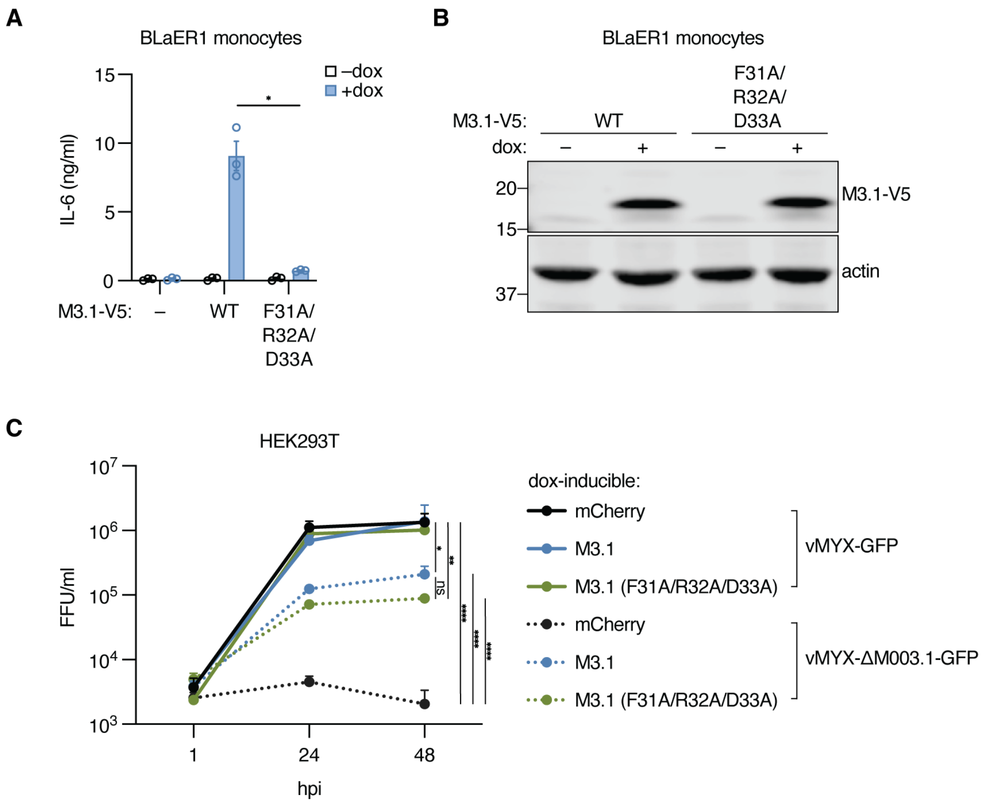

Fig. S8. The interaction between M3.1 and NYN ribonucleases is required for M3.1-mediated NF-κB signaling in BLaER1 monocytes. (**A**) Secreted IL-6 from BLaER1 monocytes treated with doxycycline for 24 hours to induce expression of mCherry (–), M3.1 (WT), or M3.1 F31A/R32A/D33A. Each data point represents an independent experiment; bars indicate mean ± SEM. (**B**) Lysates from (A) were immunoblotted as indicated. (**C**) HEK293T cells were transduced to express doxycycline-inducible mCherry, M3.1 (WT), or M3.1 F31A/R32A/D33A. Cells were treated with doxycycline and infected with MYXV (MOI = 1) for the indicated times. Data are mean ± SEM of 2-3 independent experiments. * $P < 0.05$; ** $P < 0.01$; **** $P < 0.0001$; ns = not significant, tested by unpaired t-test with Welch's correction (A) or by 2-way ANOVA with Tukey's post-hoc test on log-normalized data (C, 48 hpi).

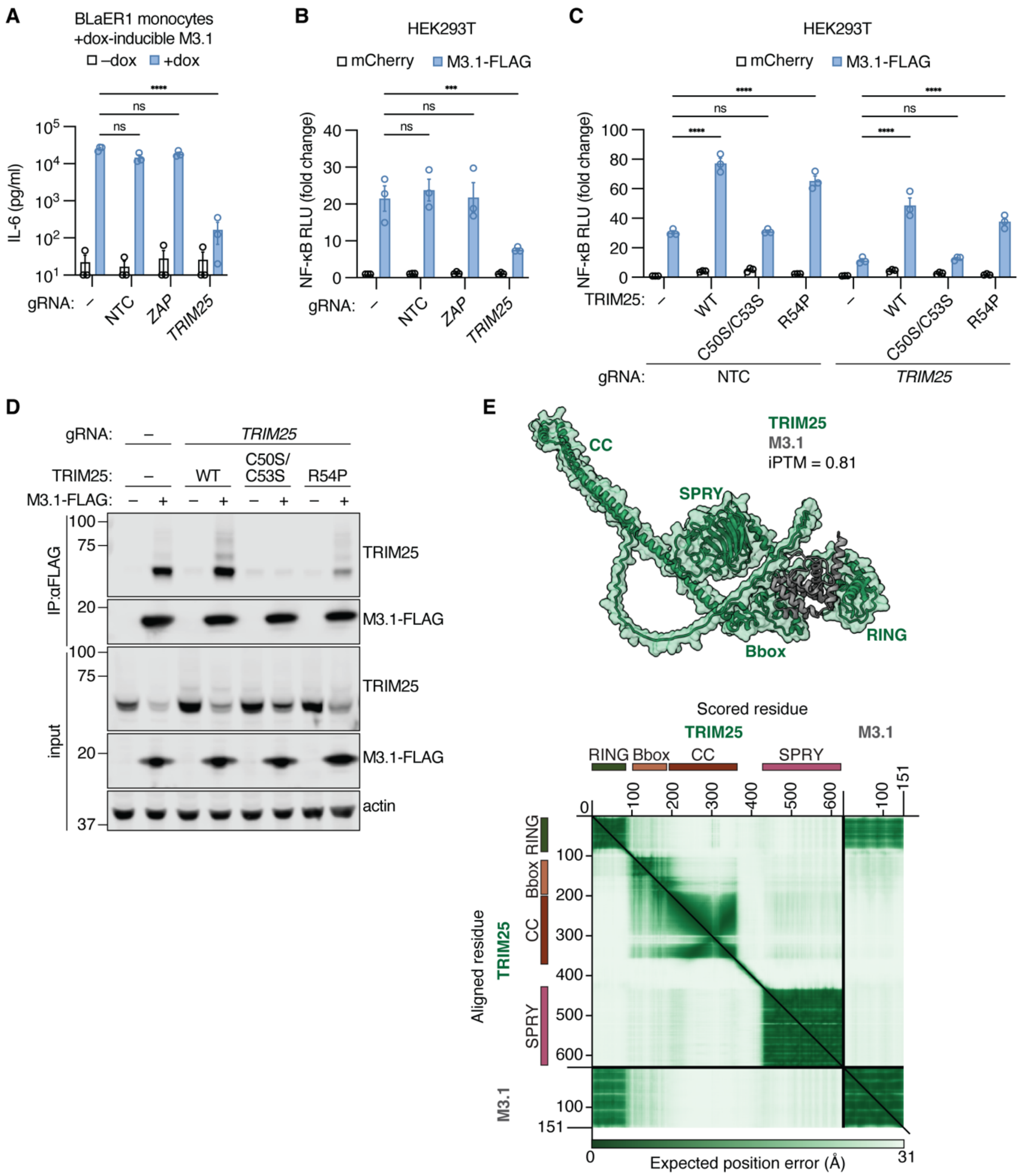

Fig. S9. TRIM25 promotes M3.1-mediated NF-κB signaling. (**A**) BLaER1 monocytes expressing doxycycline-inducible M3.1 were nucleofected with Cas9 and two gRNAs targeting the indicated gene. Cells were treated with doxycycline for 24 hours to induce expression of M3.1, and IL-6 production was measured by ELISA. Each data point represents an independent experiment; bars indicate mean ± SEM. (**B**) Cas9-RNP nucleofection was used to disrupt the indicated genes in HEK293T cells, and NF-κB induction by M3.1 was measured using a luciferase reporter. Each data point represents an independent experiment; bars indicate mean ± SEM. (**C**) A luciferase reporter was used to measure NF-κB induction in TRIM25-deficient or control HEK293T cells co-expressing TRIM25 variants and M3.1. Each data point represents an

independent experiment; bars indicate mean ± SEM. (**D**) M3.1-FLAG was immunoprecipitated from WT and TRIM25-deficient HEK293T cells expressing TRIM25 variants. The interaction between M3.1 and TRIM25 was assessed by immunoblotting. (**E**) AlphaFold 3 was used to generate the predicted structure of the complex of M3.1 and TRIM25. Corresponding PAE plot is shown. RING: Really Interesting New Gene; CC: Coiled-Coil; SPRY: SPla and the RYanodine Receptor. *** $P < 0.001$; **** $P < 0.0001$; ns = not significant, tested by 2-way ANOVA with Šídák's post-hoc test (A-C). Statistical testing for (A) was performed on log-normalized data.

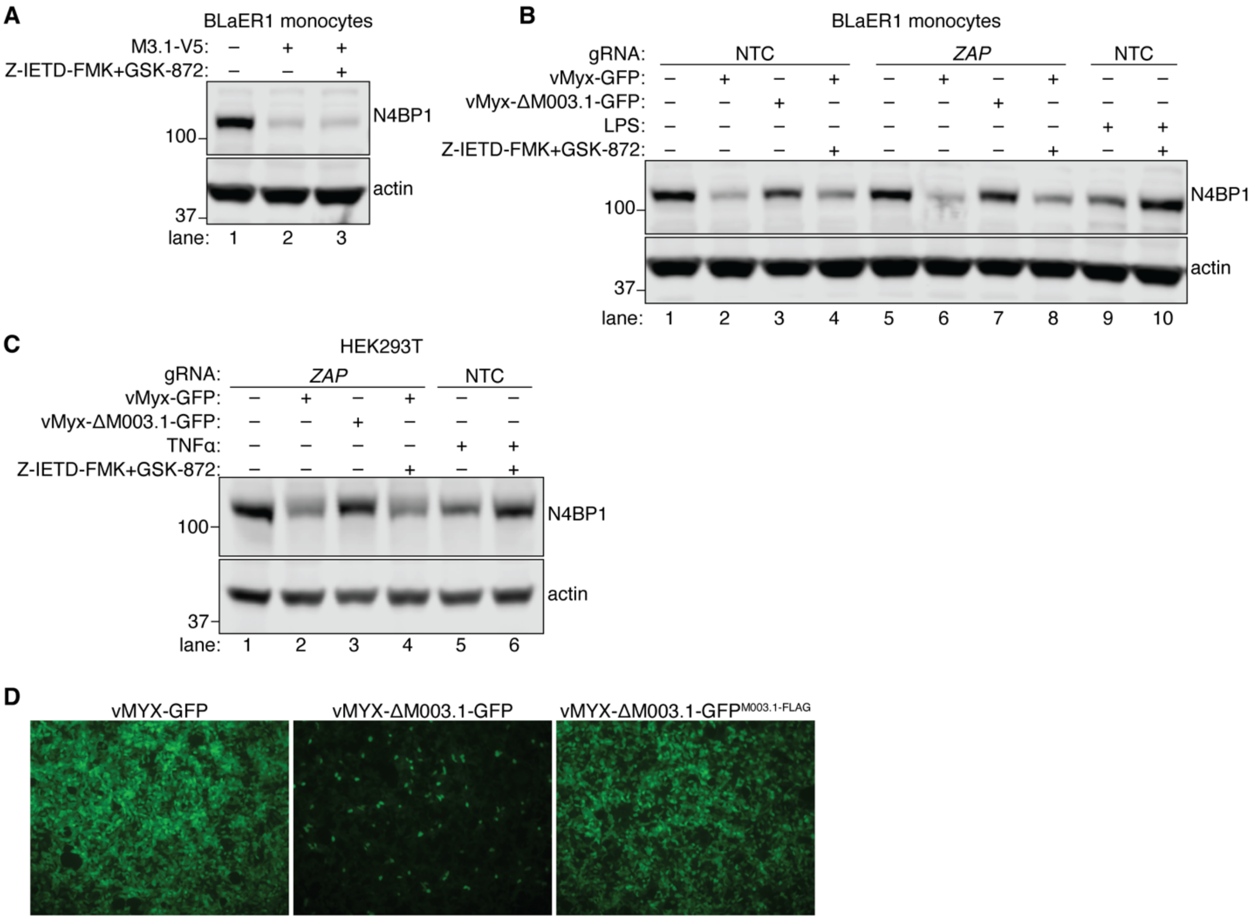

Fig. S10. M3.1 promotes caspsase-8-independent degradation of N4BP1. (**A**) BLaER1 monocytes expressing doxycycline-inducible M3.1 or mCherry were treated with doxycycline for 24 hours in the presence of caspase-8 (10 µM Z-IETD-FMK) and RIPK3 (3 µM GSK-872) inhibition or DMSO control. Lysates were immunoblotted as indicated. Images are representative of two independent experiments. (**B**) BLaER1 monocytes were either stimulated with LPS (200 ng/ml) or infected with MYXV (MOI = 3) for 24 hours in the presence of caspase-8 (10 µM Z-IETD-FMK) and RIPK3 (3 µM GSK-872) inhibition or DMSO control. Lysates were immunoblotted as indicated. Images are representative of two independent experiments. (**C**) HEK293T cells were either stimulated with TNFα (50 ng/ml) or infected with MYXV (MOI = 3) for the indicated times. Lysates were immunoblotted as indicated. (**D**) HEK293T cells were infected with the indicated MYXV strains (MOI = 3) for 24 hours. Images are representative of three independent experiments.

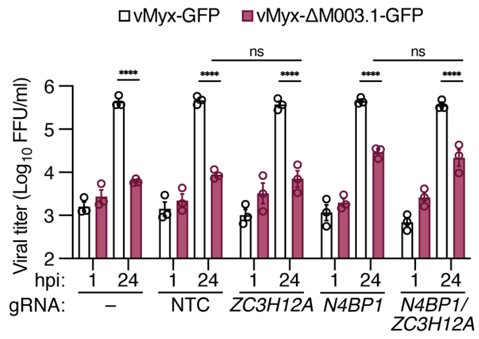

Fig. S11. ZC3H12A does not restrict MYXV. MYXV replication in HEK293T cells infected with an MOI of 1. Viral progeny were quantified at the indicated time points. Each data point represents an independent experiment; bars indicate mean ± SEM. **** $P < 0.0001$; ns = not significant, tested by 3-way ANOVA of log-normalized data with Tukey's post-hoc test.

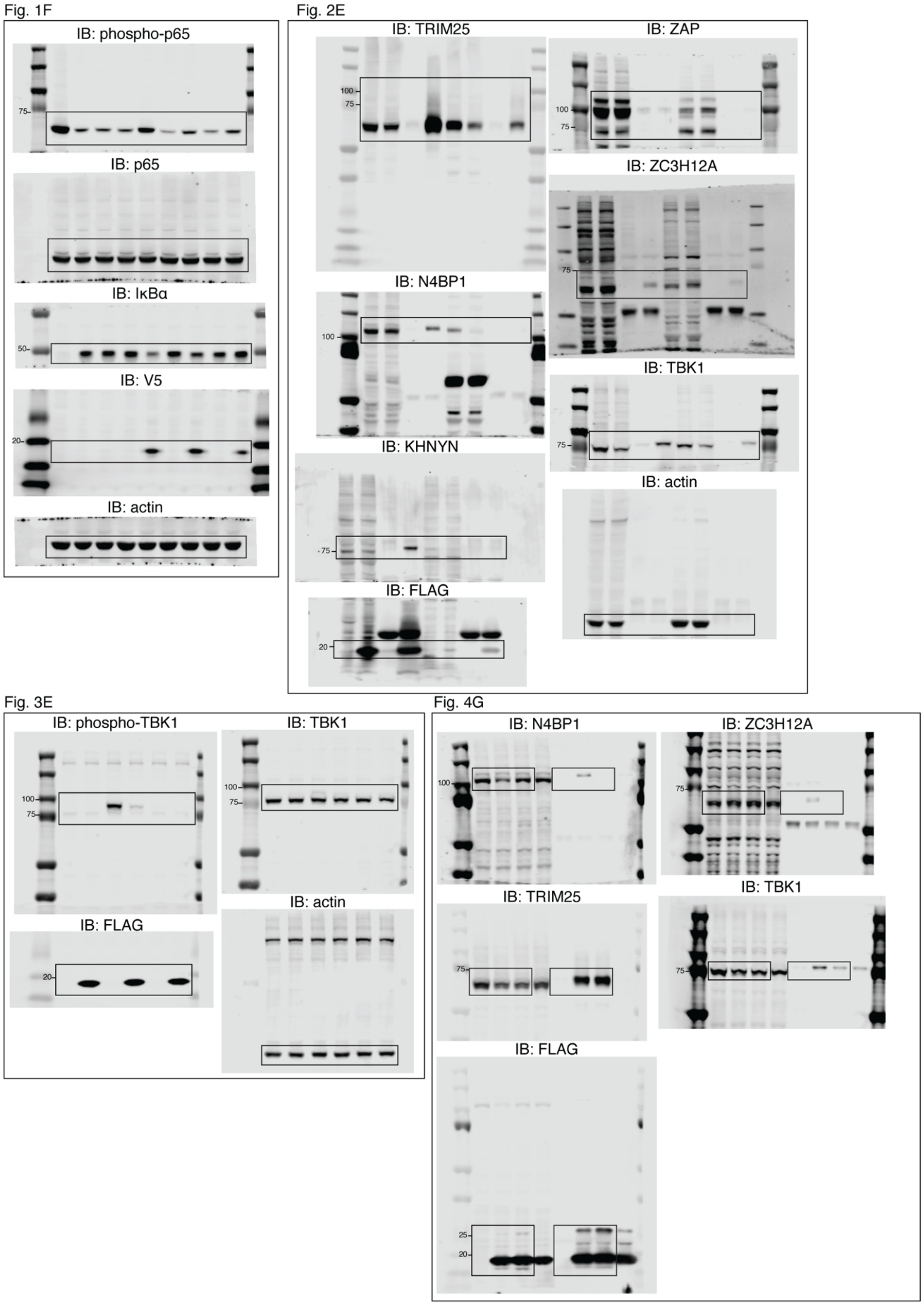

Fig. S12. Complete scans for immunoblots presented in Figures 1-4.

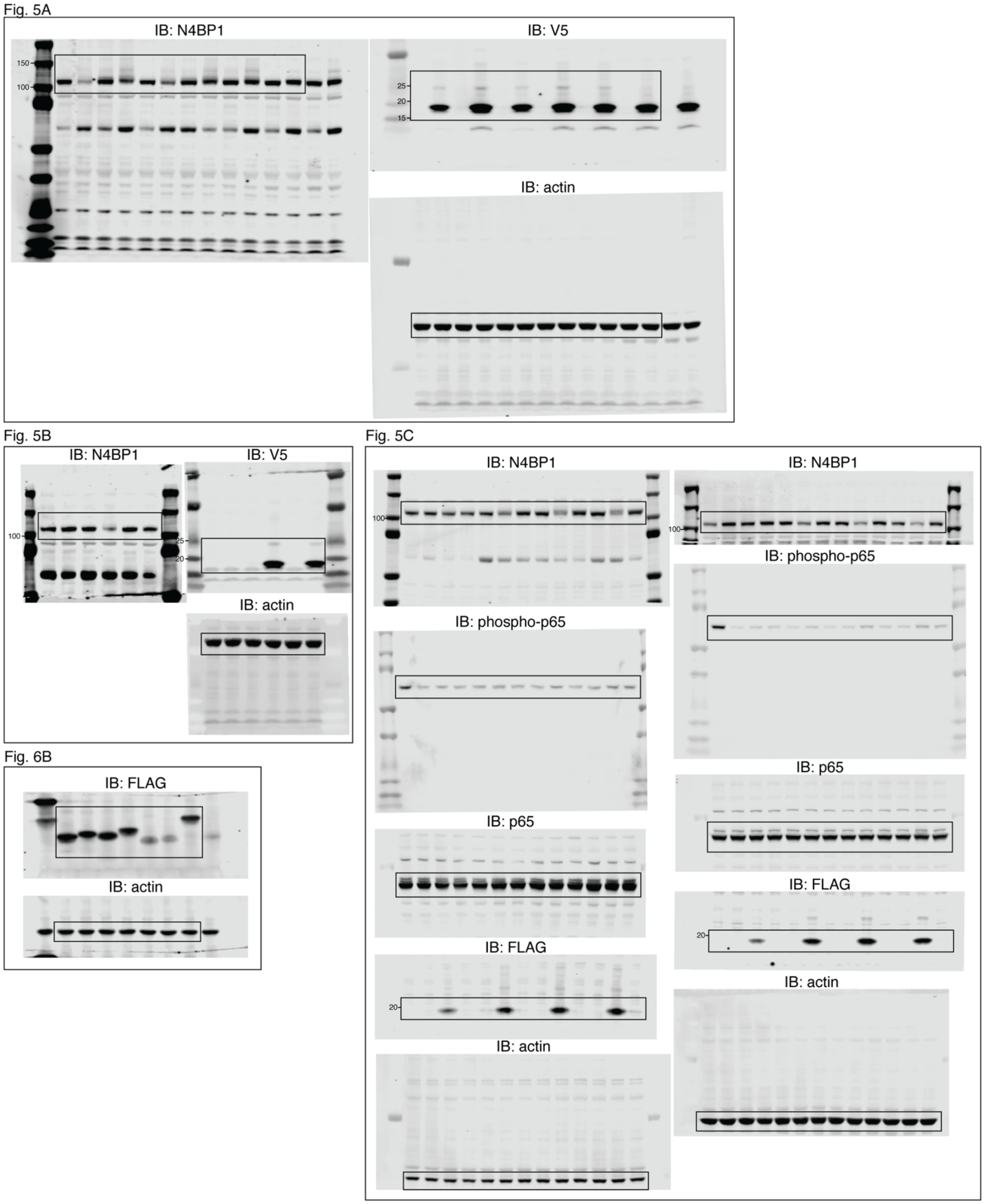

Fig. S13. Complete scans for immunoblots presented in Figures 5-6

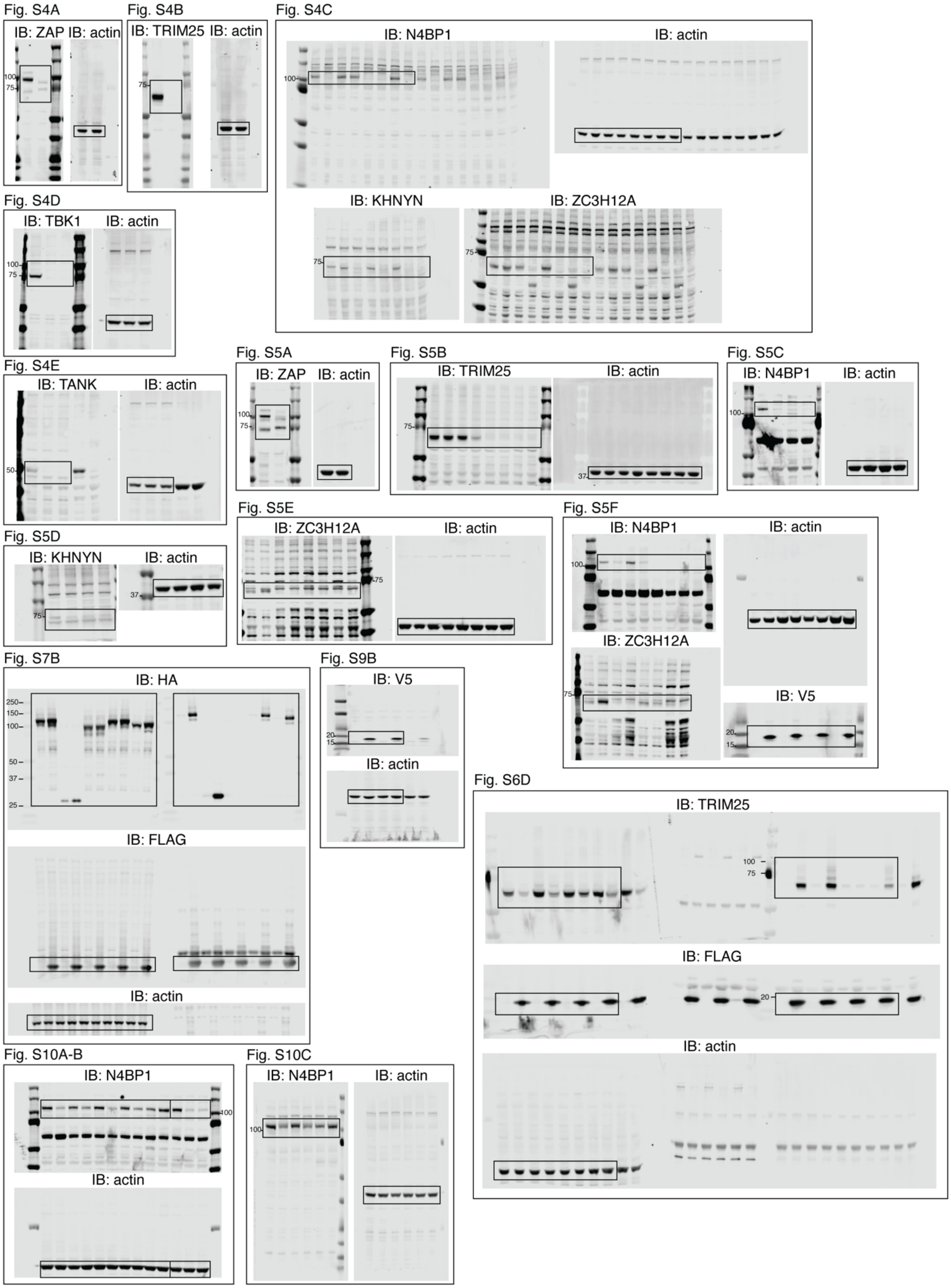

Fig. S14. Complete scans for all immunoblots presented in supplemental figures.

Data S1. (separate file) MYXV ORFs used to screen for ETI responses in BLaER1 monocytes. ORFs that were omitted from the screen due to failed transductions are indicated.

Data S2. (separate file) CompPASS results from IP-MS of M3.1-FLAG in HEK293T cells. M3.1-FLAG was immunoprecipitated from HEK293T cells that were either unstimulated or treated with TNFα (10 ng/ml) for 8 hours. M3.1 binding partners were identified by mass spectrometry followed by CompPASS analysis. Total spectral counts (TSC) were normalized to 1000 bait counts.
